# Determinants of Microbial-Derived Dissolved Organic Matter Diversity in Antarctic Lakes

**DOI:** 10.1021/acs.est.3c00249

**Published:** 2023-03-22

**Authors:** Morimaru Kida, Julian Merder, Nobuhide Fujitake, Yukiko Tanabe, Kentaro Hayashi, Sakae Kudoh, Thorsten Dittmar

**Affiliations:** † Research Group for Marine Geochemistry (ICBM-MPI Bridging Group), Institute for Chemistry and Biology of the Marine Environment (ICBM), University of Oldenburg, Carl-von-Ossietzky-Str. 9-11, Oldenburg 26129, Germany; ‡ Soil Science Laboratory, Graduate School of Agricultural Science, Kobe University, 1-1 Rokkodai, Nada, Kobe, Hyogo 657-8501, Japan; § Department of Global Ecology, Carnegie Institution for Science, 260 Panama Street, Stanford, California 94305, United States; ∥ National Institute of Polar Research, Research Organization of Information and Systems, 10-3 Midori-cho, Tachikawa, Tokyo 190-8518, Japan; ⊥ Department of Polar Science, SOKENDAI (The Graduate University for Advanced Studies), 10-3 Midori-cho, Tachikawa, Tokyo 190-8518, Japan; # Institute for Agro-Environmental Sciences, NARO, 3-1-3 Kannondai, Tsukuba, Ibaraki 305-8604, Japan; ¶ Helmholtz Institute for Functional Marine Biodiversity (HIFMB) at the University of Oldenburg, Oldenburg 26129, Germany

**Keywords:** carbon cycling, DOM, fresh waters, mass spectrometry, nuclear magnetic resonance spectroscopy, photodegradation

## Abstract

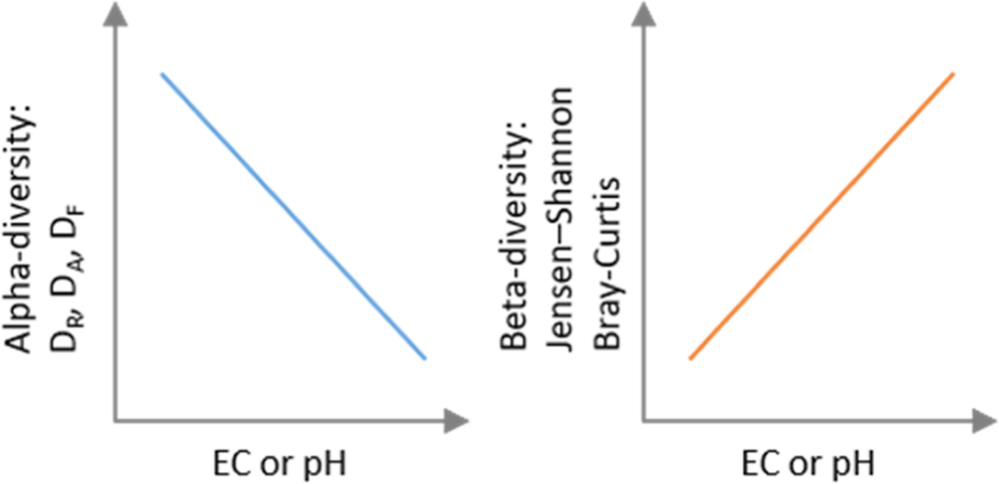

Identifying drivers of the molecular composition of dissolved organic matter (DOM) is essential to understand the global carbon cycle, but an unambiguous interpretation of observed patterns is challenging due to the presence of confounding factors that affect the DOM composition. Here, we show, by combining ultrahigh-resolution mass spectrometry and nuclear magnetic resonance spectroscopy, that the DOM molecular composition varies considerably among 43 lakes in East Antarctica that are isolated from terrestrial inputs and human influence. The DOM composition in these lakes is primarily driven by differences in the degree of photodegradation, sulfurization, and pH. Remarkable molecular beta-diversity of DOM was found that rivals the dissimilarity between DOM of rivers and the deep ocean, which was driven by environmental dissimilarity rather than the spatial distance. Our results emphasize that the extensive molecular diversity of DOM can arise even in one of the most pristine and organic matter source-limited environments on Earth, but at the same time the DOM composition is predictable by environmental variables and the lakes’ ecological history.

## Abstract 2

Molecular diversity of DOM has recently garnered attention as its key property that influences the various roles it plays. This study identified the factors driving the microbial-derived DOM diversity in Antarctic lakes.

## Introduction

Dissolved organic matter (DOM) contains more carbon than the global marine and terrestrial biomass combined.^1^ Production and consumption processes of this vast pool of DOM are critical in aquatic biogeochemical cycles and food webs because DOM contains not only carbon but also other elements essential for life.^2^ The molecular composition of DOM is thought to play a major role in its long-term persistence in aquatic systems. Therefore, factors influencing aquatic DOM composition have been the focus of study in recent years, revealing that environmental variables such as hydrological processes and climatic factors are often the primary drivers.^3−6^

A key property of DOM is the high level of molecular diversity which can hinder microbial turnover.^7−9^ The bulk DOM consists of countless different molecules at extremely dilute concentrations, the majority of which have unknown chemical structures.^10,11^ Individual constituents of DOM may be dissolved at concentrations likely too low to compensate for the metabolic costs of their utilization and to allow encounters between specific substrate molecules and microbes that can utilize them.^7,8^ In addition to its impact on DOM persistence, the molecular diversity of DOM has broader biogeochemical significance, such as influencing microbial diversity,^12^ which may together influence ecosystem functioning.^13^ However, due to the lack of focus on molecular diversity in traditional geochemistry research,^7^ there is limited knowledge on the evolution of DOM molecular diversity in natural environments and how environmental factors shape this diversity, with only a few incubation experiments providing insights into this area (e.g., refs (9)(14), and (15)).

The numerous sources and reactions in the environment are so entangled that unambiguously interpreting observed patterns is challenging.^16^ One way to minimize confounding factors is to utilize simplified environmental settings where such factors are limited. In ice-free areas of continental Antarctica, DOM is primarily derived from in situ biological activity in water.^17,18^ In several ice-free areas on Sôya Coast of East Antarctica, hundreds of lakes with varying water chemistry [e.g., electrical conductivity (EC), pH, or dissolved organic carbon (DOC) and inorganic nutrient concentrations] are distributed.^19^ Most lakes on Sôya Coast are hydrologically disconnected,^19,20^ thus serving as excellent natural incubation experiments for studying the processes responsible for the generation and transformation of microbial-derived DOM with varying water chemistry under identical climate conditions with only limited direct human influences. Historically, it has been thought that the bulk chemical characteristics of DOM in Antarctic lakes are similar due to the simplicity of their source.^17,21^ However, our recent study in 47 lakes on Sôya Coast revealed considerable variability in the composition of the optically active portion of DOM.^20^ Therefore, it is of immediate interest to determine whether DOM in these Antarctic lakes exhibits high molecular diversity despite the simplicity of the organic matter source.

In this study, we aimed to explore the evolution of molecular diversity in DOM in lakes located in one of the most pristine and organic matter source-limited environments on Earth and to identify the factors that drive diversification. We further tested whether DOM beta-diversity increases with spatial distance or environmental dissimilarity. A previous study conducted on Sôya Coast observed that benthic mats of lakes spontaneously lifted and washed ashore, possibly contributing to cycling of matter by transporting the lake’s photosynthetic products to nearby lakes as well as the surrounding ecosystems.^22^ We therefore hypothesized that both spatial distance and environmental dissimilarity are drivers of DOM beta-diversity, as is analogous to observations in ecology,^23^ and that photodegradation as a result of prolonged water retention time plays a major role in shaping the molecular diversity of microbial-derived DOM in Sôya Coast lakes.

## Materials and Methods

### Study Area and Sampling

Water sampling was conducted in five ice-free areas named Skarvsnes (*n* = 28), Skallen (*n* = 7), Langhovde (*n* = 8), Rundvågshetta (*n* = 3), and Breivågnipa (*n* = 2), located on Sôya Coast (Lützow-Holm Bay, East Dronning Maud Land, East Antarctica), during the austral summer in December 2016 and February 2017 as a part of the 58th Japanese Antarctic Research Expedition.^20,24^ Most lakes are sustained solely by meltwater from adjacent glaciers and/or snow and ice within the catchments. A layer of ice approximately 1–2 m thick typically forms on these lakes except during the brief austral summer period. Consequently, water sampling was restricted to this seasonal window. Previous year-round monitoring of limnological parameters in Skarvsnes^25^ has revealed clear seasonal patterns in freshwater lakes. Water temperature, turbidity, and photosynthetically active radiation are the highest in summer, and chlorophyll *a* concentration reaches its minimum due to intense light inhibiting phytoplankton growth. The maximum chlorophyll *a* concentration is typically observed under the dim-light conditions of spring and autumn.^25^ It is currently unknown whether similar seasonal patterns exist in saline or hypersaline lakes.

The sources of organic matter in these lakes are presumably benthic mats consisting primarily of algae, cyanobacteria, and mosses, rather than phytoplankton which are present in low abundance.^26^ The level of benthic mat development varies among lakes, being the least developed in proglacial lakes, dominated by cyanobacteria in the anoxic bottom of hypersaline lakes, and being abundant in some lakes for currently unspecified reasons. The influence of migratory birds (*Pagodroma nivea* and *Stercorarius maccormicki*) on these lakes is generally minimal. The prevailing wind direction is from the continental ice shelf to the coasts (katabatic wind), possibly limiting the aerial transport of matter between lakes in that direction (i.e., from lakes located closer to the glacier to those located farther) and minimizing the influence of marine aerosols to the lakes.

Lake water was collected at half the depth of the water column from a boat using a Teflon cylindrical water sampler into 550 mL volume polyethylene terephthalate bottles after rinsing more than three times with the collected water. In all freshwater lakes, the water column was completely mixed according to uniform water chemistry by a multi-water-quality logger (YSI-6600V2, YSI Inc., OH, USA) measuring depth, temperature, pH, EC, turbidity, dissolved oxygen, and oxidation–reduction potential. Saline lakes with water stratification were sampled at several depths to capture changes in water chemistry. When water samples were collected from shore, samples were collected directly into the storage bottles and the pH, EC, and water temperature were recorded in situ using portable water quality meters (LAQUA series, Horiba, Kyoto, Japan). Sample handling was performed as previously reported;^20^ briefly, the collected waters were filtered with a precombusted glass fiber filter (GF-75, ADVANTEC, Kyoto, Japan; nominal pore size of 0.3 μm) in a field laboratory within a few hours of sampling into acid-cleaned muffled glass bottles with a PTFE liner and stored in the dark at 4 °C until analysis. The sampling locations, limnological characteristics, water chemistry, and optical and chemical properties of bulk DOM for each lake are provided in Table S1 and previously summarized and thoroughly described.^20^

There exist several marine relict lakes that were previously a part of the coastal marine shelf and formed following the iso-static uplift of the present lake shores after the Last Glacial Maximum. The majority of marine relict lakes contain freshwater, yet some relict lakes formed below sea level are hypersaline because they are terminal without outflow and materials captured within are trapped unless they precipitate or decompose. Based on the deviation from the conservative mixing line between EC and DOC, we calculated the possible contributions of legacy marine DOM to contemporary DOM in hypersaline lakes (Figure S1).

### Water Analysis

UV–vis absorption spectra and fluorescence excitation-emission matrix spectra were measured within one week after filtration as previously reported.^20^ DOC-specific ultraviolet absorbance (SUVA_254_, in L mg C^–1^ m^–1^) and spectral slope (*S*_275–295_, in nm^–1^) were derived.^20^ Seven underlying fluorescence components were previously identified by parallel factor analysis.^20^ DOC and total dissolved nitrogen were determined in the laboratory in Japan using a total organic carbon analyzer combined with a total nitrogen measuring unit (TOC-L_CPH_, Shimadzu, Kyoto, Japan).^27^ Dissolved inorganic nutrients (PO_4_^3–^, NH_4_^+^, NO_2_^–^, NO_3_^–^, and SiO_3_^2–^) were determined colorimetrically using an autoanalyzer (AACS III AutoAnalyzer, Bran + Luebbe). Dissolved organic nitrogen (DON) was calculated by the difference between total dissolved nitrogen and the sum of inorganic nitrogen species (DIN).

Bulk water ^1^H NMR with the SPR-W5 WATERGATE sequence^28^ was conducted on a Bruker AVANCE 500 spectrometer (Bruker GmbH, Karlsruhe, Germany) with a 5 mm double resonance broadband (BBI) probe using 5 mm Shigemi symmetrical susceptibility-matched NMR tubes (BMS-005B, Shigemi, Tokyo, Japan), as previously described.^24,29^ This sequence achieves very high sensitivity for low-abundant DOC samples by effectively deleting the water signal and maximizing the receiver gain, with a slight attenuation of signals up to 1.1 ppm on either side of the water resonance.^28^ Only 35 samples could be analyzed due to sample limitation. To facilitate NMR analysis, samples corresponding to 0.05–0.1 mg of C were evapo-concentrated using small pear-shaped flasks and a rotary evaporator at <40 °C and transferred to NMR tubes with D_2_O after filtering with precleaned glass fiber syringe filters (Whatman GF/F) using a glass syringe with a Teflon plunger tip.^24^ Typically, NMR spectra were acquired with 128–512 scans, shaped 180° pulse (P20) of 4 ms, a binomial delay (D19) of 155 μs, and 36,406 time domain points with an acquisition time of 3.29 s. Samples from hyper saline lakes (Lakes Suribachi and Funazoko) and a saline lake (Lake Nurume) were analyzed without pre-concentration to avoid loss of DOC through co-precipitation with salts. For these samples, 512–11000 scans were collected depending on sample DOC concentrations, making analysis time ranging between 1 h up to overnight. Spectra were calibrated to trimethylsilyl resonance (0 ppm) of sodium 3-trimethylsilylpropionate-2,2,3,3-*d*_4_ (TMSP-d4, Euriso-top, Saint-Aubin, France) and apodized by multiplication with an exponential decay, producing a 2 Hz line broadening in the transformed spectrum.

### DOM Extraction and Fourier Transform Ion Cyclotron Resonance Mass Spectrometry Measurements

After acidification (pH 2) by 25% HCl, DOM was extracted and desalted for Fourier transform ion cyclotron resonance mass spectrometry (FT-ICR MS) analysis following the established method^30^ using Agilent Bond Elut PPL (100 mg) cartridges with a slight modification regarding sample preparation and DOC loading. Depending on the sample’s DOC concentration, the volume corresponding to 4 μmol of C was subsampled. Furthermore, subsamples were diluted to a fixed volume (85 mL) using ultrapure water before extraction. Extracting the same DOC amount and volume of samples (and hence the same DOC concentration) minimized possible artifacts during DOM extraction by PPL. The methanol extracts were stored at −20 °C in the dark. The average extraction efficiency was, on average, 35% ± 10% on a DOC basis and positively correlated with a relative abundance of the hydrophobic fraction of DOM^20^ (% HPO, *r* = 0.48), while it negatively correlated with a relative abundance of protein-like fluorescence^20^ (*r* = −0.68). The relatively low extraction efficiency, especially in lakes dominated by proteinaceous materials, indicated a recent microbial source and the lack of strong microbial processing of DOM in the studied lakes.^24^ The extracted DOM, referred to hereafter as solid-phase extracted DOM (SPE-DOM), was within the analytical window of FT-ICR MS.

We performed mass spectrometric analysis of SPE-DOM on a 15 Tesla solariX XR FT-ICR mass spectrometer (Bruker Daltonik GmbH, Bremen, Germany), equipped with an electrospray ionization source (ESI, Bruker Apollo II) applied in the negative ionization mode, and a PAL autosampler. Before analysis, the extracts were diluted to a final concentration of 2.5 mg C L^–1^ in a mixture of methanol and ultrapure water (1:1 v/v). A total of 200 transients in a scanning range of 92–2000 Da were co-added for each sample with an ion accumulation time of 0.1 s. At each day of extraction, a process blank extract was prepared by processing 85 mL of pH 2 ultrapure water (0.01 M HCl) the same way as the samples. An in-house reference material extracted using PPL from North Equatorial Pacific Intermediate Water^31^ was analyzed under the identical settings, to test the instrument reproducibility and stability. Molecular formulae (MFs) of detected masses were assigned as previously described^24^ (details can be found in S1). In total, 9910 MFs were used for further analysis. Subsequently, the modified aromaticity index (AI_mod_),^32^ degradation index (*I*_Deg_),^33^ as well as molar elemental ratios were calculated for each formula. MFs were grouped into four descriptive compound categories, namely, aromatic (AI_mod_ > 0.5), highly unsaturated (AI_mod_ ≤ 0.5 & H/C < 1.5), unsaturated (H/C ≥ 1.5 & double bond equivalent, DBE ≠ 0), and saturated (DBE = 0) MFs. All four categories were subdivided into high- (O/C ≥ 0.5) and low-oxygen (O/C < 0.5) content formulas.

### Molecular Alpha- and Beta-Diversity of DOM

All statistical analysis was conducted using R statistical language.^34^ As indicators for molecular alpha-diversity, we calculated the molecular richness *D*_R_, the abundance-based Gini-Simpson index (Simpson index of diversity) *D*_A_, and the functional molecular diversity *D*_F_ of SPE-DOM analyzed by FT-ICR MS according to Mentges et al.^35^ Molecular richness *D*_R_ was defined as the number of MFs identified in a sample by FT-ICR MS. *D*_R_ is a simple measure of molecular alpha-diversity of DOM, but replicate measurements of the same sample can vary considerably.^36^ The Gini-Simpson index (or Simpson’s index of diversity) *D*_A_ applied to FT-ICR MS data uses the relative peak intensity distribution, and the value can be interpreted as the probability that the MFs of two randomly chosen molecules differ. The index ranges between 0 and 1, where larger values indicate higher diversity. The functional diversity *D*_F_ applied to FT-ICR MS data is computed as a distance function (Rao’s quadratic entropy) using the absolute difference between any two MFs with respect to a given chemical property, weighted by their relative peak intensities. The value of the functional diversity can be interpreted as the expected difference between two molecules with respect to the selected property.^35^ In this study, the functional diversity was calculated with the number of C atoms as indicators of molecular weight, hydrogen-to-carbon (H/C) ratio as an indicator of saturation, nitrogen-to-carbon (N/C) ratio as an indicator of relative nitrogen richness, the AI_mod_^32^ and DBE as indicators of DOM aromaticity, and nominal oxidation state of carbon (NOSC)^37^ as an indicator of the average oxidation state of MFs which has been related to compound reactivity. The oxidation of an organic compound becomes thermodynamically more profitable as NOSC increases.

The Jensen–Shannon divergence (JSd),^38^ a dissimilarity used in genetics and information theory, was used to compute molecular beta-diversity (dissimilarities) between samples. The JSd compares the frequency distributions of selected MF characteristics between samples (i.e., intensity information was not used) and is bound between 0 and 1 when using the binary logarithm in its calculation. These characteristics can be, for example, the relative frequencies of compound classes or the distribution of MF-derived indices such as DBE_AI_. DBE_AI_ is the numerator of AI_mod_ and is calculated as the DBE of the resulting molecular core after all functional groups that potentially contribute DBE through bonds between carbon and heteroatoms are subtracted from the original MF.^32^ Because DBE_AI_ is naturally binned in 0.5 intervals, the empirical probability for each bin can be easily calculated from a sample so that DBE_AI_ is preferred over AI_mod_ for JSd computation and used here. In addition, we found that JSd based on DBE_AI_ achieved better clustering (fewer samples with a negative silhouette value) and identified more meaningful indicator species than classical Bray–Curtis dissimilarities based on relative peak intensities.

For the Mantel test described below, molecular beta-diversity of SPE-DOM was computed as Bray–Curtis dissimilarity of 9910 normalized FT-ICR MS peak intensities, which allows for direct comparison with previous studies that used Bray–Curtis dissimilarities. Peak intensities for each sample were normalized to the sum of peak intensities for that sample.

We note that any measures of DOM molecular diversity based on MFs or peak intensities underestimate its true molecular diversity, as there are numerous isomers behind a given MF or peak. However, with current analytical techniques, it is impossible to derive chemical structures for all MFs in the data set. The MF approach is practical and conservative and can be readily used for calculating diversity indices across studies.

### Statistical Analysis

Non-metric multidimensional scaling (NMDS) and hierarchical clustering with average linkage agglomerative clustering^39^ were performed based on the JSd. An optimal number of clusters was found by maximizing the silhouette coefficient and comparison between the dissimilarity matrix and binary matrices representing group allocations.^39^ External variables were fitted post-hoc to the NMDS ordination space^40^ with *p*-values calculated over 9999 permutations. MF characteristics for each cluster were calculated using indicator species values (IndVal),^41^ the product of the relative frequency and relative average abundance of MFs in a pre-defined group (cluster in this case).^42^ This index is maximum (i.e., 1) when a given MF is found in a single cluster and when an MF is found in all lakes in that cluster. The statistical significance of the indicator values was tested by 9999 permutations. The permutational *p*-value for each MF was adjusted for multiple testing,^43^ and an adjusted *p*-value ≤ 0.05 was considered significant. Average chemical properties were calculated for each cluster, regarding the contents of nitrogen and sulfur within MFs, mass, AI_mod_, and DBE_AI_. Environmental variables that could explain the clustering were identified by a conditional inference tree (CTree)^44^ (Figure 4). A CTree uses repeated binary data splits at thresholds of exploratory variables (environmental variables) so that between-cluster separation is maximized.

We also conducted PCA on ^1^H NMR data to evaluate if NMDS on FT-ICR MS data sufficiently captured bulk DOM compositional differences. This was particularly relevant because FT-ICR MS was conducted on a relatively small portion of PPL-extracted DOM (SPE-DOM). The signal intensities for the NMR spectra at 0.5–4.4 ppm were exported for each sample at a resolution of 0.005 ppm (780 data points per sample). Aromatic signal regions were not included because of the lack of signals in most samples. PCA was performed after NMR data were scaled to unit variance (i.e., correlation matrix), otherwise few sharp signals from small molecules would dominate the loadings. Environmental variables were post-hoc fitted as in NMDS.

Finally, we tested whether spatial distance or environmental dissimilarity can each explain molecular beta-diversity of SPE-DOM. The influences of these factors on SPE-DOM beta-diversity (Bray–Curtis dissimilarity) were assessed by the Mantel test.^40,45^ This test compares two similarity/dissimilarity matrices calculated for the same objects and tests a hypothesis about the relationship between them.^46^ Here, we tested the hypothesis that beta-diversity of SPE-DOM increases with either spatial distance or environmental dissimilarity. Spatial distance among lakes was calculated as the great-circle distance between lakes on a sphere, given their longitudes and latitudes.^47^ Environmental dissimilarity was computed as the Euclidean distance of the standardized (*z*-scored) environmental variables. Mantel statistic (*r*_M_), which is a Pearson correlation between entries of the two dissimilarity matrices, was derived after standardizing the values in each matrix.^46^ The significance of the Mantel statistic was tested by 9999 permutations. Environmental variables used for the Mantel test were selected using the Bayesian information criterion (BIC)^48^ for a beta regression model^49^ with Bray–Curtis dissimilarity as a response variable and the Euclidian distances between the *z*-scored environmental variables as explanatory variables. In addition, we estimated the partial effects of the BIC-selected environmental variables on Bray–Curtis dissimilarities.^50^ We also estimated the partial effects of BIC-selected environmental variables on alpha-diversity in the same manner.

## Results

### Molecular Properties of Antarctic Lake DOM: Alpha-Diversity

**Figure 1:**
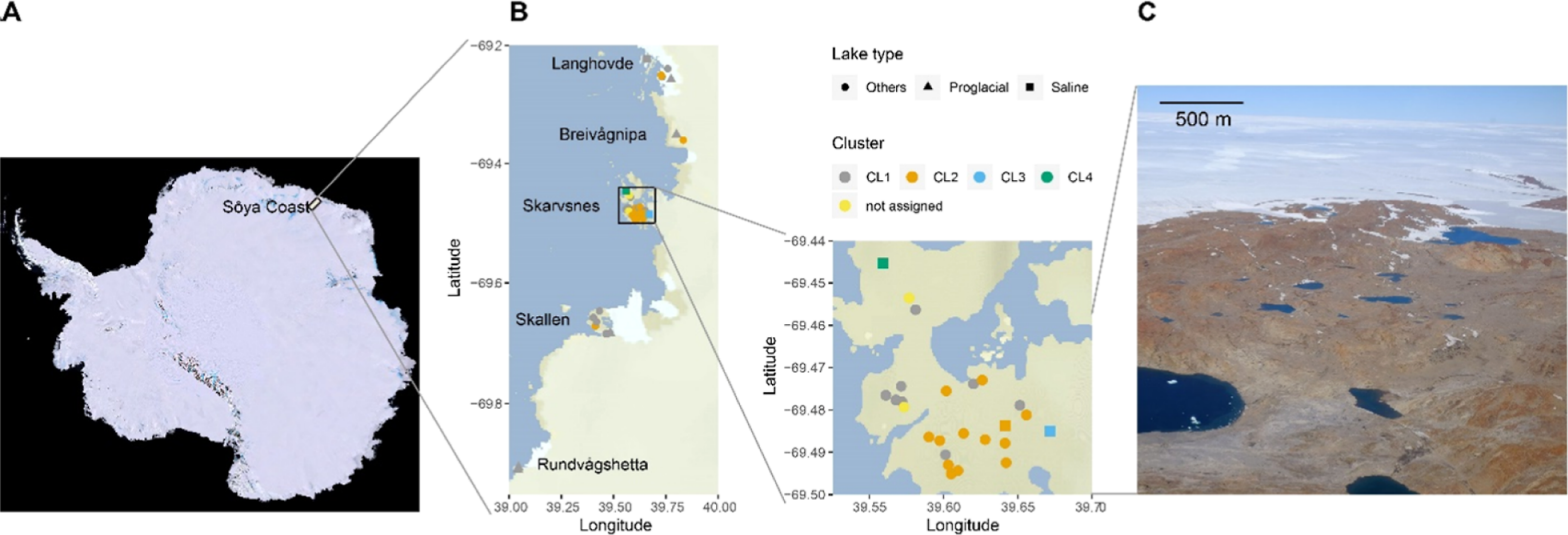
Lakes in Antarctic ice-free areas provide an excellent natural experimental field to study diverse microbial-derived DOM. (A) Overview of Antarctica with the study site (Sôya Coast, Lützow-Holm Bay, and East Dronning Maud Land) highlighted, (B) Spatial distribution of sampled lakes in five ice-free areas on Sôya Coast, with different symbols representing the lake types and colors representing the clusters identified based on DOM molecular data (Figure S7). The area marked with the rectangle (Skarvsnes) in the left tile is shown in the right tile, and (C) aerial photograph of one of the study sites (Skarvsnes), showing lakes separated by impermeable bed rocks.

EC of the lakes ranged very broadly from freshwater to hypersaline (>22 S m^–1^) (Figure S1). The pH of the lakes was generally neutral to slightly basic, with values ranging from 7.15 to 8.86. We observed an exponential increase in DOC with EC (ranging from 0.25 to 146 mg C L^–1^), indicating increased in situ net production as well as evapo-concentration in the lakes with increasing EC (Figure S1). In the hypersaline marine relict lakes (clusters 3 and 4 in Figure 1), we conservatively estimated that at most only 1.5–7.6% of the DOC present could be legacy marine DOC, given that there has been no decomposition of legacy DOM for approximately 7000 years since the formation of these lakes.^19^ Therefore, we ruled out the contribution of legacy marine DOM in all of the studied lakes. A very low molecular degradation index (*I*_Deg_)^33^ of 0.15 ± 0.03 also supports the conclusion that DOM in these lakes is relatively young.

**Figure 2:**
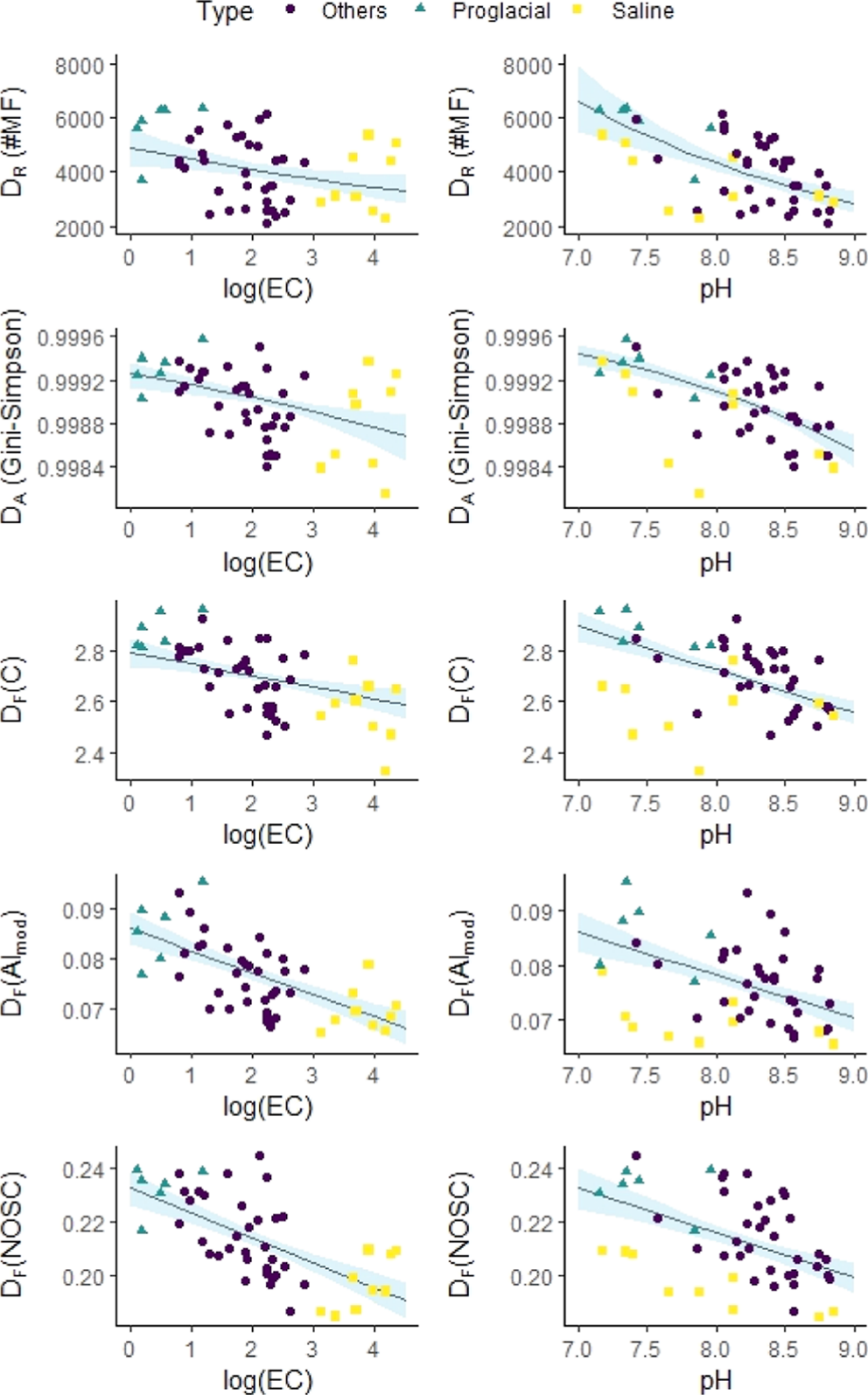
Electrical conductivity (EC, log-scale) and pH are predictors of alpha-diversity of DOM. Alpha-diversity is quantified by molecular richness *D*_R_, abundance-based Gini-Simpson index (Simpson index of diversity) *D*_A_, as well as functional diversity regarding the number of C atoms *D*_F_(C), modified aromaticity index *D*_F_(AI_mod_),^32^ and nominal oxidation state of carbon *D*_F_(NOSC).^37^ The blue shaded areas indicate the 95% confidence intervals of the partial effects of EC and pH on alpha-diversity, as estimated via a multivariate regression. Raw data points are overlaid and colored according to the lake type.

We identified a total of 9910 unique MFs in SPE-DOM by FT-ICR MS. The molecular richness *D*_R_, or the number of MFs in each sample, varied from 2121 to 6338 (Figure 2). The van Krevelen diagram (O/C versus H/C plot) of each sample is provided in Figure S2. A decreasing number of MFs was associated with higher pH and EC (Figure 2). When accounting for covarying variables, pH, EC, and lake surface area were each significant predictors of *D*_R_ (Figure S3). A higher number of MFs tended to be detected in SPE-DOM samples from proglacial lakes where freshly produced microbial-derived biomolecules predominated.^20,24^ An increase in pH of about two units had a considerable impact on decreasing *D*_R_ to half (from approximately 6000 to 3000, Figure 2). EC similarly decreased *D*_R_ when accounting for other variables (Figure 2). The abundance-based diversity D_A_ similarly decreased with increasing pH and EC, and again, pH, EC, lake surface area, and altitude were significant predictors (Figure S3). All series of functional diversity *D*_F_ also clearly decreased with increasing EC and pH (Figures 2, S4, and S5).

### Drivers of Beta-Diversity

**Figure 3:**
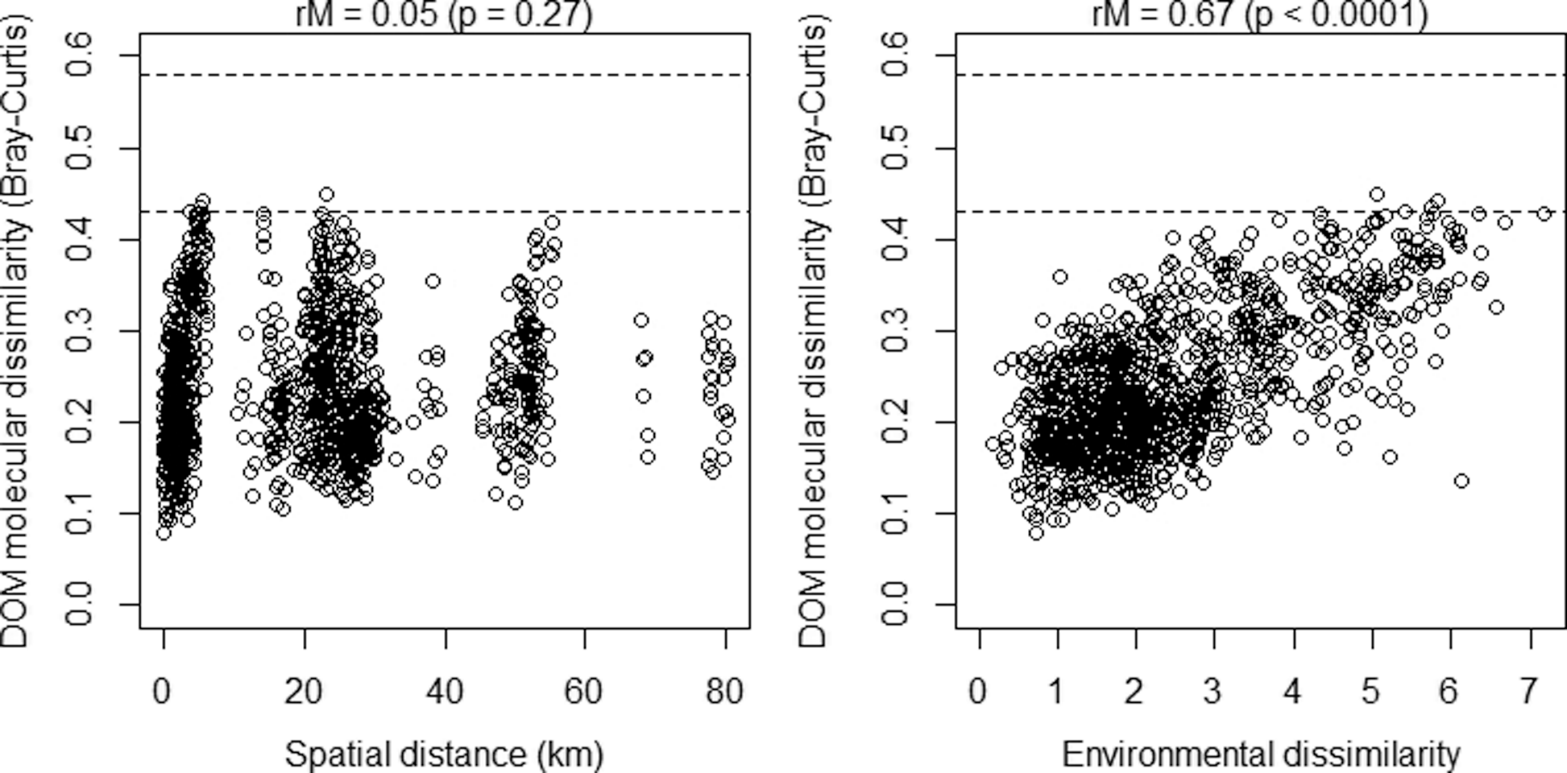
Environmental dissimilarity, not spatial distance, between lakes is a driver of DOM molecular dissimilarity (beta-diversity). *r*_M_ = Mantel statistic, where the significance was tested by 9999 permutations. DOM molecular beta-diversity was computed as Bray–Curtis dissimilarity of 9910 normalized FT-ICR-MS peak intensities. The considered environmental variables include water chemistry (electrical conductivity, pH, and temperature) and nutrient abundance (DIN), which were identified as significant drivers of DOM beta-diversity (Figure S6). The dashed lines indicate the Bray–Curtis dissimilarity ranges between DOM of 10 world rivers and North Equatorial Pacific Intermediate Water analyzed by FT-ICR-MS (Riedel et al. 2016). Bray–Curtis dissimilarity of the same sample can vary by up to 0.05 in replicate analyses.

We tested if spatial distance or environmental dissimilarity can each explain SPE-DOM beta-diversity using the Mantel test.^45^ SPE-DOM beta-diversity, computed as Bray–Curtis dissimilarity of 9910 normalized FT-ICR MS peak intensities, ranged between 0.08 and 0.46 (Figure 3). The environmental variables particularly responsible for the observed SPE-DOM beta-diversity were identified via beta regression (Figure S6)^51^ and used for calculating the overall environmental dissimilarity. We found that spatial distance was not a driver of SPE-DOM diversity at our observed scale (Figure 3). In contrast, environmental dissimilarity, calculated as the Euclidean distance of standardized (*z*-scored) environmental variables, was a strong influencing factor of beta-diversity (Figure 3). Dissimilarities of EC, water temperature, pH, and DIN concentrations were selected as all significantly influencing SPE-DOM beta-diversity (Fig. S6).

### Grouping of Lakes Based on DOM Molecular Properties

The molecular differences between the samples were represented in two-dimensional space via non-metric multidimensional scaling (NMDS) based on the Jensen–Shannon divergence (JSd)^38^ which compares the frequency distributions of a molecular index known as DBE_AI_ (see the Materials and Methods section) (Figure S7). Hierarchical clustering indicated that the samples could most appropriately be divided into four groups according to the JSd (Figures S8 and S9). The spatial distribution of the samples is illustrated in Figure 1. The first cluster (CL1) was present in all areas and included all of the proglacial lakes and was characterized by smaller lake and catchment areas, lower levels of evapo-concentration (as indicated by lower values of EC, *a*_254_, DOC, DIN, and DON), and a high relative abundance of humic-like components and aromaticity (as measured by a humic-like fluorescence component C_500_ and SUVA_254_)^20^ (Figure S7). The second cluster (CL2) contained lakes other than hypersaline lakes and was primarily found not only in Skarvsnes but also in other areas (Figure 1). The third and fourth clusters (CL3 and CL4) consisted of two hypersaline lakes (at two depths each) in Skarvsnes and were associated with larger lake and catchment areas, a higher degree of photodegradation (as indicated by higher *S*_275–295_),^20^ and elevated values of quantitative parameters indicative of greater evaporation (Figure S7). Post-hoc fitting of environmental variables revealed that pH was the only characteristic that clearly distinguished CL1 and CL2 (Figure S7). Therefore, FT-ICR MS detected differences in SPE-DOM composition between CL1 and CL2 that were not readily discernible through other water chemical characteristics and bulk DOM properties.

**Figure 4:**
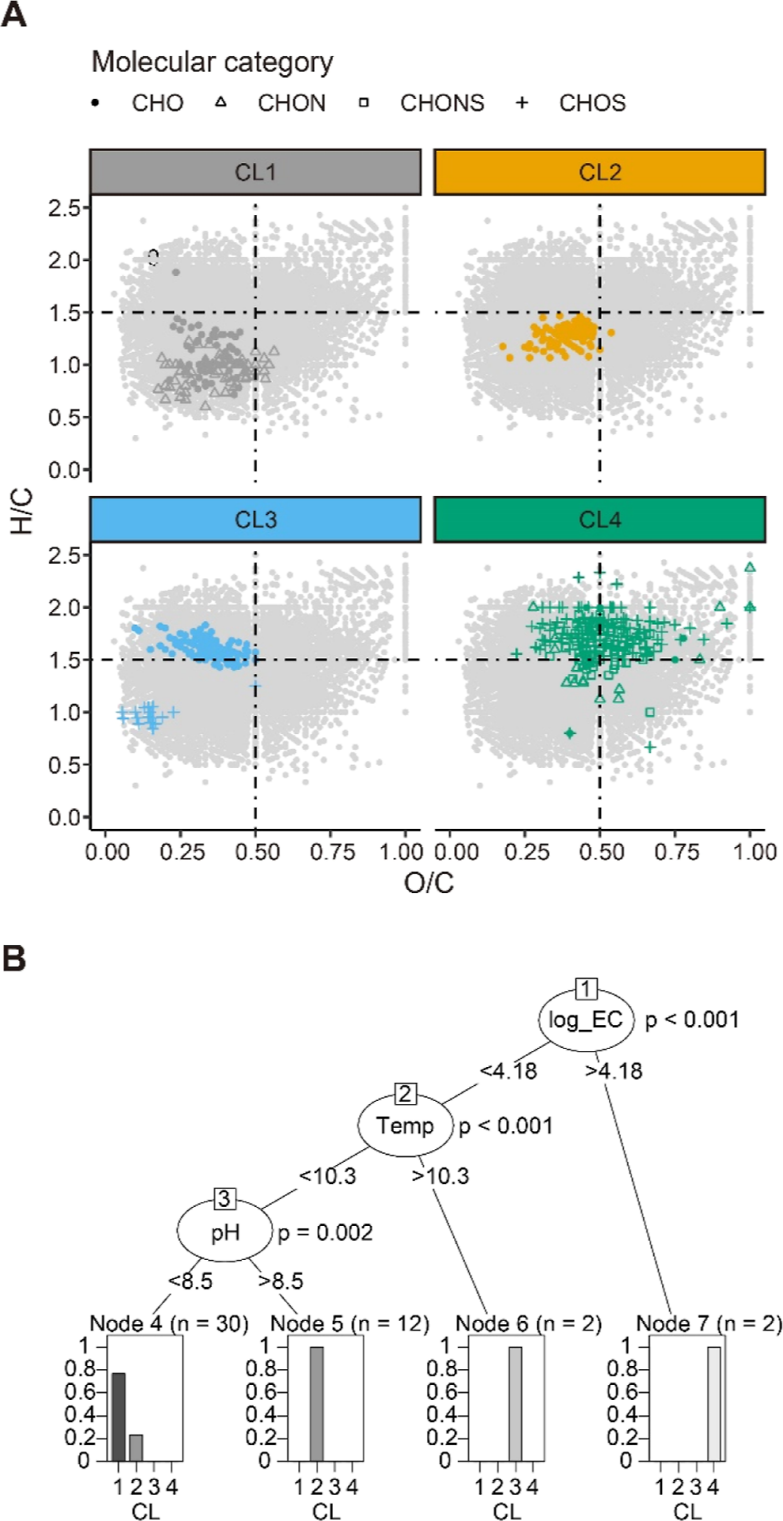
Antarctic lakes develop distinct molecular properties, and the differences are well explained by environmental variables. (A) Indicator molecular formulas (MFs) that are significantly associated with each cluster. MFs are plotted in the van Krevelen space with different shapes representing different heteroatoms in each MF. Background gray dots are all considered molecular formulae (*n* = 9910). The dotted lines indicate the boundary between unsaturated and highly unsaturated MFs (H/C = 1.5) and relative O contents (O/C = 0.5). CL = cluster. (B) Conditional inference tree (CTree) identifying environmental variables that discriminated the clusters. For each inner node (represented by circles), the Bonferroni-adjusted *p*-values are given, while the fraction of lakes assigned to each cluster is displayed for each terminal node (represented by rectangles).

We identified key MFs that were particularly associated with each cluster (Figure 4), using the IndVal index which is commonly used in ecology.^39^ A summary of MFs selected as indicators of each cluster based on their IndVal values is provided in Table S2. No P-containing MFs appeared as indicators. The first cluster CL1 (proglacial lakes) was associated with O-poor (O/C < 0.5), highly unsaturated (AI_mod_ ≤ 0.5 and H/C < 1.5), and aromatic (AI_mod_ > 0.5) MFs, which were N-rich and S-free. The second cluster CL2 was exclusively associated with heteroatom-free, highly unsaturated MFs. The third cluster CL3 (hypersaline lake Suribachi) was enriched in both unsaturated (H/C ≥ 1.5) and highly unsaturated/aromatic MFs. The highly unsaturated/aromatic MFs in CL3 almost all contained one S, while the unsaturated MFs were heteroatom-free. The fourth cluster CL4 (hypersaline lake Funazoko) was enriched in unsaturated MFs. Many of the unsaturated MFs of CL4 contained a variable number of N and S. The relative abundance of S or N containing MFs for indicator species of each cluster is provided in Figure S10, while the distribution of mass, AI_mod_, and DBE_AI_ are presented in Figure S11. The indicator MF of each cluster exhibited distinct mass and AI_mod_ distribution, although differences in the latter are expected, as the molecular index used for calculating JSd is the numerator of AI_mod_.^32^ The mass distribution followed CL2 ∼ CL4 > CL1 ∼ CL3, while the AI_mod_ distribution was clearly CL1 > CL2 > CL3 > CL4 (Figure S11). These features of the indicator MF were consistent with differential van Krevelen diagrams of proglacial and hypersaline lakes (Figure S12). MFs detected in only proglacial lakes were mainly O-poor, highly unsaturated, and aromatic MFs with at least one N, while those unique in surface water of the hypersaline lakes were S-rich unsaturated MFs and some S-rich highly unsaturated/aromatic MFs (Figure S12). Using a conditional inference tree (CTree), environmental variables that could best explain the clustering were identified (Figure 4). CL4 was first separated by high EC, and CL3 was separated by water temperature. CL1 and CL2 were distinguished by pH (Figure 4).

We conducted a principal component analysis (PCA) on bulk water ^1^H NMR data to determine whether NMDS on FT-ICR MS data (Figure S7) adequately captured the compositional differences in bulk DOM. We confirmed that key features of the two ordination results were similar; CL1 and CL2 were well separated on the ordination space, and proglacial lakes and hyper saline lakes were plotted on the edge of the sample distribution cloud (Figure S13). Representative bulk DOM NMR spectra are presented in Figure S13. Principal component 1 (PC1) discriminated samples based on the degree of microbial processing of DOM, where more processed DOM with broad signals^24^ were located on the positive end of PC1, while more “fresh”, less processed DOM with sharp signals derived from small biomolecules^24,52^ were located on the negative end of PC1 (Figure S13). PC2 separated samples based on the relative abundance of carbohydrate H. Carbohydrate-rich DOM was associated with positive PC2, while aliphatic- or functionalized aliphatic-rich DOM^24^ was associated with negative PC2 (Figure S13). Water pH still stands out as a distinguishing factor between CL1 and CL2, together with altitude. Quantitative parameters such as DOC, DON, and *a*_254_ again failed to explain differences between these two clusters.

## Discussion

### Drivers of DOM Alpha-Diversity: Photodegradation and pH Variation

In the Antarctic lakes studied, EC and pH each decreased all the three types of SPE-DOM alpha-diversity (Figures 2, S4, and S5). This is surprising because other environmental variables such as water temperature and nutrients (DIN, PO_4_^3–^, and SiO_3_^2–^) that presumably strongly impact microbial activities did not covary with these parameters.^20^ The evapo-concentration process in these lakes occurs at variable rates, reflecting the balance between prevailing evaporation under dry conditions and freshwater inputs from catchment snow and ice. Typically, a higher EC value indicates a lower net freshwater gain and thus longer water retention time.^20^ This longer water retention time in turn leads to photodegradation of DOM, as indicated by a clear correlation between the spectral slope (*S*_275–295_), an index of photodegradation, and EC.^20,53^ We acknowledge the need for cautious employment of *S*_275–295_ as a photodegradation index, as it has also been linked to differences in the organic matter source and molecular weight.^54^ However, for this specific data set, our prior research has demonstrated its suitability as a photodegradation index,^20^ partly because of simplicity of source and a clear environmental gradient in a degree of photodegradation. There were also strong negative correlations between an increasing degree of photodegradation (*S*_275–295_) and aromatic proxies for the optically active portion of DOM (Figure S7). Apparently, enhanced photodegradation of DOM due to the longer water retention time selectively diminished light-absorbing, aromatic, and/or heteroatom-containing compounds^20,53,55^ (Figure S12), which did not decrease bulk DOC (Figure S1). The selective degradation of certain compounds decreased *D*_R_ and resulted in a less uniform abundance distribution of compounds and decrease in *D*_A_ (Figure 2), while it also decreased *D*_F_ regarding aromaticity (H/C, AI_mod_, and DBE) (Figures 2 and S4). The ranges of *D*_F_ were much closer to SPE-DOM data from a 3 year incubation study where artificial sea water was inoculated with coastal North Sea water microbial communities, rather than a field observation in the Atlantic and Southern Ocean in which the ages of DOM were estimated to be thousands of years,^15,35^ suggesting that DOM in the Antarctic lakes was at the initial stage of the degradation cascade.

The decrease in SPE-DOM alpha-diversity with increasing pH (Figure 2) was likely caused by abiotic factors. The slightly basic pH of the studied lakes is due to the bedrock composition which is mostly pyroxene or garnet-biotite gneiss. Weathering of such a bedrock releases Ca and Mg, leading to an increase in lake water pH. Variations in pH were, however, not associated with other water constituents, lake morphology, or bedrock composition (pyroxene versus garnet-biotite gneiss) and may reflect weathering degrees of the bedrock in the lakes. High pH can lead to co-precipitation of the most oxidized DOM compounds with iron and aluminum hydroxides,^56^ thereby reducing the set of alpha-diversities. Additionally, an increase in pH could enhance the photodegradation efficiency of DOM because of deprotonation of carboxyl and phenolic groups and/or conformational changes^57^ and may result in a decrease in alpha-diversity with increasing pH through enhanced photodegradation.

### Drivers of DOM Beta-Diversity: Spatial Distance versus Environmental Dissimilarity

Surprisingly, the organic matter source-limited Antarctic lakes were very dissimilar in terms of their SPE-DOM molecular composition (Bray–Curtis dissimilarity ranging from 0.08 to 0.46 and mean 0.25 ± 0.08, Figure 3). In contrast, SPE-DOM from ten of the largest world rivers spanning from the Tropics to the Arctic was molecularly more similar (0.17 ± 0.06)^4^ to each other. SPE-DOM between some of the lakes even showed dissimilarities as great as those observed between rivers and the deep ocean (Figure 3). How can such a large beta-diversity emerge among lakes that are located within a spatially limited area under identical climate? To approach this question, we first tested the hypothesis that spatial distance is related to SPE-DOM beta-diversity. However, we observed the opposite; at the spatial scale we examined, spatial distance had no effect on SPE-DOM beta-diversity. Specifically, SPE-DOM from lakes that were in close proximity to each other was molecularly not more similar compared to SPE-DOM from distant lakes (Figure 3). This finding is contrary to a paradigm in theoretical ecology, which posits that community composition similarity is expected to decrease with distance,^23^ refuting our first hypothesis. The lack of a significant relationship between SPE-DOM beta-diversity and distance indicates that beta-diversity of SPE-DOM may be driven by small-scale environmental heterogeneity that does not vary consistently with distance. This is consistent with the fact that most lakes are hydrologically disconnected and have developed over thousands of years^19^ under lake-specific settings that are stochastically determined by the surrounding environment (such as lake morphology, geology, microclimate, and biological activities within the catchments). Accordingly, we found a significant and strong correlation between environmental dissimilarity (pH, EC, temperature, and DIN) and SPE-DOM molecular diversity (*r*_M_ = 0.67) (Figure 3). While microbes residing in the lakes likely play a major role in shaping SPE-DOM diversity as they do in other aquatic settings,^12,13^ we lack the microbial data to test that. Nevertheless, since microbial composition and function are largely driven by the environment, environmental dissimilarity would hold as a strong driver of SPE-DOM molecular diversity in the studied lakes.

The dissimilarity in EC was the most influential parameter on the SPE-DOM beta-diversity (Figure S6). SPE-DOM composition likely diverged under varying degrees of evapo-concentration and photodegradation among the lakes. Water temperature (at the time of sampling) was the second strongest influential parameter (Figure S6). Water temperature may indirectly influence SPE-DOM composition through changes in microbial activity, but in our data set, it was more likely due to exceptional water temperature in the hypersaline lakes (12–21 °C in Lake Suribachi and −3.5 to 20 °C in Lake Funazoko)^20^ and the distinct DOM molecular compositions in these lakes (Figures 4 and S7). SPE-DOM in the hypersaline, marine relict lakes contained a high abundance of *S* likely because of abiotic sulfurization of DOM by sulfide under the anoxic condition caused by water stratification^20,58^ (Figures 4 and S10). The high relative abundance of *S* even in the surface water of the hypersaline lakes indicates that there was a net production of *S*-containing DOM (DOS) in spite of extensive photodegradation in these lakes.^20,59^ A recently proposed refractory nature of DOS^58^ may contribute to the extensive accumulation of DOM in the hypersaline lakes. High DIN values were also observed in the hypersaline lakes, making them an apparent driver of SPE-DOM beta-diversity (Figure S6). Water pH also had an impact on SPE-DOM beta-diversity (Figure S6). Lower pH values were observed in proglacial lakes of which SPE-DOM was particularly enriched in N (Figures 4, S10, and S12). The enrichment of N in SPE-DOM in proglacial lakes is consistent with the previous findings that DOM in meltwater from glaciers on Sôya Coast is enriched in labile N-containing compounds.^24^ The relative enrichment of N in proglacial lakes indicates that, although all of the samples in this study are primarily of microbial origin, the relative N abundance can vary between samples depending on the “freshness” of DOM. These N-containing labile compounds are presumably rapidly consumed by heterotrophic bacteria along with other small biomolecules after glacial melt, leading to the production of more complex DOM and enhancing DOM molecular diversity.^9,24^

In summary, we show that both alpha- and beta-diversity of SPE-DOM are predictable based on only a few primary water chemistry parameters (Figures 2 and S3–S6). Additionally, our results indicate that the composition of SPE-DOM becomes more unique to individual lakes as water retention time increases and as alpha-diversity decreases with elevated EC, while beta-diversity increases with greater EC differences (i.e., proglacial lakes and hypersaline lakes display the greatest dissimilarity in terms of SPE-DOM composition) (Figures 2 and S3–S6). This molecular succession of DOM over time and resulting specificity of DOM composition serve as an excellent proxy for the lakes’ ecological history.

## Data Availability

An R code for the Jensen-Shannon divergence to compute dissimilarities of the distribution of molecular indices between samples is available at Zenodo (doi: 10.5281/zenodo.6944776). FT-ICR MS data will be freely available at PANGAEA (doi: 10.1594/PANGAEA.956291).

## Supporting Information Available

Description about FT-ICR MS data processing, summary of basic sample information, relationship between electrical conductivity and dissolved organic carbon concentrations, van Krevelen diagrams of all SPE-DOM samples, estimated partial effects of environmental variables on alpha-diversities, partial effects of electrical conductivity and pH on functional diversity measures, beta regression results showing estimated partial effects of environmental dissimilarity on DOM molecular beta-diversity, multivariate analysis of dissolved organic matter molecular data using non-metric multidimensional scaling, determination of an optimal number of clusters, relative abundance of S- or N-containing MF and distributions of mass, AI_mod_, and DBE_AI_ for indicator species of each cluster, synthetic van Krevelen diagrams of SPE-DOM of proglacial lakes and hypersaline lakes and their differences, and PCA of bulk water ^1^H NMR signal intensities, with representative NMR spectra (PDF)
Summary of MFs selected as indicators of each cluster based on their IndVal values (PDF)
