# Supplementary material for: Determinants of Microbial-Derived Dissolved Organic Matter Diversity in Antarctic Lakes
Source: Environ Sci Technol. 2023 Mar 22;57(13):5464–73. doi: 10.1021/acs.est.3c00249 (PMC10077579; doi:10.1021/acs.est.3c00249)
Supplement: Supplementary file 1 — es3c00249_si_001.pdf [file es3c00249_si_001.pdf]

Determinants of microbial-derived dissolved organic matter diversity in Antarctic lakes

Morimaru Kida[1,2]*, Julian Merder[3], Nobuhide Fujitake[2]*, Yukiko Tanabe[4, 5], Kentaro Hayashi[6, †], Sakae Kudoh[4, 5], Thorsten Dittmar[1,7]*

[1] Research Group for Marine Geochemistry (ICBM-MPI Bridging Group), Institute for Chemistry and Biology of the Marine Environment (ICBM), University of Oldenburg, Carl-von-Ossietzky-Str. 9-11, Oldenburg 26129, Germany

[2] Soil Science Laboratory, Graduate School of Agricultural Science, Kobe University, 1-1 Rokkodai, Nada, Kobe, Hyogo 657-8501, Japan

[3] Department of Global Ecology, Carnegie Institution for Science, 260 Panama Street, Stanford, CA 94305, USA

[4] National Institute of Polar Research, Research Organization of Information and Systems, 10-3 Midori-cho, Tachikawa, Tokyo 190-8518, Japan

[5] Department of Polar Science, SOKENDAI (The Graduate University for Advanced Studies), 10-3 Midori-cho, Tachikawa, Tokyo 190-8518, Japan

[6] Institute for Agro-Environmental Sciences, NARO, 3-1-3 Kannondai, Tsukuba, Ibaraki 305-8604, Japan

[7] Helmholtz Institute for Functional Marine Biodiversity (HIFMB) at the University of Oldenburg, Oldenburg 26129, Germany

† Current address

Research Institute for Humanity and Nature, 457-4 Motoyama, Kamigamo, Kita, Kyoto, 603-8047, Japan

* corresponding author

morimaru.kida@people.kobe-u.ac.jp

fujitake@kobe-u.ac.jp

thorsten.dittmar@uni-oldenburg.de

Number of pages: 16

Number of tables: 1

Number of figures: 12

S1 FT-ICR MS data processing

Mass spectra were internally recalibrated with a list of 74 known $C_xH_yO_z$ molecular formulae (MF) covering the mass range of the samples using Bruker Daltonics Data Analysis software. Calibrated mass lists were processed with a freely available mass spectra processing tool, ICBM-OCEAN [1] (Beta-Version 01-Dec-2019). Detected masses were matched across all samples and molecular formulae assigned to the final mass list, allowing a mass error of <0.5 ppm [2]. MF were assigned following Koch et al. (2007), with maximum elemental abundances of $C_{100}H_{200}O_{70}N_6S_2P_1$ and a tolerance range of 0.5 ppm. MF that include all the heteroatoms simultaneously were not considered. All samples were run twice, and the mass spectra reported here represent the mean intensities of the replicates. Singly detected peaks in replicate measurements were removed to increase confidence in formula assignment. After merging replicates, all MF found in only one sample across the dataset (n = 48) were further removed. Peaks detected at least once in the process blanks (n = 11) with a signal intensity > 10 × method detection limit [4] were considered contaminations and excluded. Isotopologues (MF containing ^{13}C, ^{18}O, ^{15}N, ^{34}S) were also excluded from the dataset. MF with double bond equivalents (DBE) minus the number of O atoms of greater than 10 were removed [5].

References

(1) Merder, J.; Freund, J. A.; Feudel, U.; Hansen, C. T.; Hawkes, J. A.; Jacob, B.; Klaproth, K.; Niggemann, J.; Noriega-Ortega, B. E.; Osterholz, H.; et al. ICBM-OCEAN: Processing ultrahigh-resolution mass spectrometry data of complex molecular mixtures. *Anal. Chem.* **2020**, *92* (10), 1–12.

(2) Merder, J.; Freund, J. A.; Feudel, U.; Niggemann, J.; Singer, G.; Dittmar, T. Improved mass accuracy and isotope confirmation through alignment of ultrahigh-resolution mass spectra of complex natural mixtures. *Anal. Chem.* **2020**, *92* (3), 2558–2565.

(3) Koch, B. P.; Dittmar, T.; Witt, M.; Kattner, G. Fundamentals of molecular formula assignment to ultrahigh resolution mass data of natural organic matter. *Anal. Chem.* **2007**, *79* (4), 1758–1763.

(4) Riedel, T.; Dittmar, T. A method detection limit for the analysis of natural organic matter via Fourier transform ion cyclotron resonance mass spectrometry. *Anal. Chem.* **2014**, *86* (16), 8376–8382.

(5) Herzsprung, P.; Hertkorn, N.; von Tümpling, W.; Harir, M.; Friese, K.; Schmitt-Kopplin, P. Understanding molecular formula assignment of Fourier transform ion cyclotron resonance mass spectrometry data of natural organic matter from a chemical point of view. *Anal. Bioanal. Chem.* **2014**, *406* (30), 7977–7987.

Table S1. The sampling locations, limnological characteristics, and basic optical and chemical properties of DOM for each lake of Lützow-Holm Bay ice-free area.

Area name	Lake name	Water type	Altitude	Area	Max. depth	Sampling depth	Sampling date	EC	pH	Temp	$SUVA_{254}$	$S_{275-295}$	DOC	%HPO	SPE-DOC recovery
			(m)	($\times 10^4$ m^2)	(m)	(m)		(mS m^{-1})		(℃)	(L mgC^{-1} m^{-1})	(nm^{-1})	(mg C L^{-1})	(%)	(%)
B	Hiroe	proglacial	215	35.2	9.5	4.0	19 Jan. 2017	1.5	7.84	2.9	0.71	0.020	0.36	46.5	27.4
B	Breivagnipa Lake1	proglacial	285	0.6	ND	shore	19 Jan. 2017	41	7.86	6.4	0.65	0.033	1.36	36.0	36.6
L	Yukidori	others	125	4.1	8.6	3.5	10 Jan. 2017	6	8.22	6.6	0.34	0.031	0.89	48.0	25.2
L	Mitsuike Ue	others	40	0.2	1.0	shore	13 Jan. 2017	122	8.36	7.8	0.68	0.029	1.67	32.1	29.9
L	Mitsuike Naka	others	40	0.2	1.0	shore	13 Jan. 2017	158	8.24	8.2	0.60	0.033	1.22	29.2	32.9
L	Mitsuike Shita	others	30	0.2	1.5	shore	13 Jan. 2017	174	8.39	8.2	0.70	0.034	2.22	31.0	35.2
L	Nurume	saline	0	3.5	16.2	3.0	12 Jan. 2017	4502	8.13	6.6	0.69	0.037	2.24	40.7	50.6
L	Nurume	saline	0	3.5	16.2	8.5	12 Jan. 2017	4926	8.13	3.2	0.73	0.033	6.21	40.7	47.3
L	Nurume	saline	0	3.5	16.2	14	12 Jan. 2017	7854	7.17	2.1			11.2	48.4	41.9
L	Heito	proglacial	165	4.9	4.5	shore	9 Jan. 2017	3.6	7.32	1.8	0.80	0.022	0.64	31.0	13.8
R	Maruwan-Oike	proglacial	10	25.2	37	9.0	17 Jan. 2017	1.3	7.96	3.6			0.56	20.4	13
R	Maruwan Kita	proglacial	15	4.6	ND	shore	17 Jan. 2017	1.5	7.44	2.3	0.44	0.028	1.02	18.3	12.1
R	Maruwan Minami	proglacial	15	2.0	11.5	shore	17 Jan. 2017	3.1	7.15	5.2	0.28	0.025	0.72	27.6	21.2
Sl	Skallen-Oike	others	5	22.5	9.2	3.5	4 Jan. 2017	27.4	8.06	4.1	0.78	0.031	1.43	48.4	40.7
Sl	Skallen Lake1	others	30	1.3	ND	shore	4 Jan. 2017	14.4	8.14	6.5	0.82	0.019	0.71	40.5	24.7
Sl	Skallen Lake2	others	30	0.3	ND	shore	4 Jan. 2017	9.6	8.40	8.6	1.07	0.019	0.78	30.1	25.8
Sl	Skallen Lake3	others	55	0.9	ND	shore	5 Jan. 2017	15.9	8.50	6.0	0.65	0.028	1.25	35.9	34.8
Sl	Skallen Lake4	others	75	1.2	ND	shore	5 Jan. 2017	7.4	8.48	9.5	0.41	0.028	0.71	32.0	22.6
Sl	Skallen Lake5	others	45	0.6	ND	shore	5 Jan. 2017	6	8.22	10.1	1.04	0.017	1.07	37.6	27.4
Sl	Glacial Lake	proglacial	10	4.6	ND	shore	4 Jan. 2017	15	7.35	7.7			0.25	41.1	37.3
Sr	Oyako	others	5	6.5	8.0	4.0	27 Dec. 2016	74.5	8.27	5.7	0.87	0.026	1.19	54.2	30.8
Sr	Naga	others	70	4.8	10.8	5.0	28 Dec. 2016	174	8.56	4.9	0.29	0.033	3.51	36.1	31.9
Sr	Nyorai	others	130	0.6	3.0	1.5	29 Dec. 2016	67.8	8.30	7.1	0.55	0.033	1.54	44.6	31.9
Sr	Hotoke	others	120	0.4	3.0	1.0	30 Dec. 2016	85.9	8.31	4.8	0.67	0.034	1.08	40.5	38.3
Sr	Kuwai	others	160	0.3	4.3	2.0	30 Dec. 2016	309	7.57	10.3	0.55	0.040	0.94	38.3	42.9
Sr	Bosatsu	others	130	0.8	4.8	2.0	25 Jan. 2017	37.5	8.05	8.4	0.47	0.032	1.38	38.4	28

Area name	Lake name	Water type	Altitude	Area	Max. depth	Sampling depth	Sampling date	EC	pH	Temp	$SUVA_{254}$	$S_{275-295}$	DOC	%HPO	SPE-DOC recovery
			(m)	($\times 10^4$ m^2)	(m)	(m)		(mS m^{-1})		(°C)	(L mgC^{-1} m^{-1})	(nm^{-1})	(mg C L^{-1})	(%)	(%)
Sr	Jizo	others	120	0.3	3.1	1.5	25 Jan. 2017	239	8.54	8.2	0.74	0.026	1.46	41.4	41.9
Sr	Himago	others	−5	0.4	ND	shore	27 Jan. 2017	710	8.42	7.8	0.85	0.035	5.23	43.2	51.8
Sr	Mago	others	−5	1.1	1.7	shore	27 Jan. 2017	420	8.56	7.3	0.76	0.035	4.57	46.3	43
Sr	Sara	others	0	1.1	2.5	1.5	27 Jan. 2017	74.7	8.75	7.1	0.95	0.031	2.09	45.0	41.8
Sr	Tokkuri	others	45	1.9	6.0	shore	28 Jan. 2017	173	8.04	6.5	0.50	0.036	2.77	48.6	24.5
Sr	Kumogata	others	65	3.2	0.8	shore	28 Jan. 2017	130	7.42	6.3	0.47	0.032	3.90	37.6	30.2
Sr	Hyotan	others	75	5.3	12.4	6.0	31 Jan. 2017	210	8.80	5.9	0.39	0.045	2.69	42.6	29.7
Sr	Hamagiku	others	95	1.9	4.0	2.0	31 Jan. 2017	12.8	8.05	4.2	0.64	0.028	0.90	38.5	29
Sr	Ebi	others	50	2.2	6.3	3.0	31 Jan. 2017	167	8.81	5.3	0.43	0.045	2.94	40.3	44.4
Sr	Kikuno	others	55	1.3	2.5	shore	31 Jan. 2017	240	8.53	4.9	0.61	0.041	1.75	36.9	44.5
Sr	Hashioki	others	10	1.2	2.0	shore	27 Jan. 2017	55	8.42	7.3	0.80	0.033	1.73	39.8	43.1
Sr	Skarvsnes Lake2	others	55	0.4	ND	shore	31 Jan. 2017	190	8.56	5.8	0.53	0.040	2.57	41.6	31.5
Sr	A-5	others	90	0.2	2.0	shore	31 Jan. 2017	80	8.59	5.1	0.69	0.038	3.36	38.6	42.2
Sr	Hechima	others	85	1.4	6.3	shore	6 Feb. 2017	320	8.73	5.2			1.43	44.8	45.1
Sr	Magobachi	others	60	1.5	5.0	shore	6 Feb. 2017	200	8.83	5	0.72	0.040	1.27	44.9	41.2
Sr	Misumi	others	90	0.7	6.3	shore	6 Feb. 2017	19	8.17	5	0.35	0.033	1.79	54.3	34.1
Sr	Kobachi	saline	25	4.4	12	1.5	27 Jan. 2017	1285	8.86	7.2	0.56	0.046	4.39	42.9	52.3
Sr	Kobachi	saline	25	4.4	12	6.0	27 Jan. 2017	2256	8.75	9.7	0.56	0.043	6.62	43.9	49.6
Sr	Suribachi	saline	−33	58.8	32	2.0	27 Jan. 2017	9572	7.66	11.8	0.22	0.068	12.2	39.6	38.1
Sr	Suribachi	saline	−33	58.8	32	6.0	27 Jan. 2017	15179	7.88	20.7	0.22	0.068	35.1	39.7	37.7
Sr	Funazoko	saline	−23	13.1	9.2	2.0	28 Jan. 2017	18307	7.40	20.3	0.45	0.053	92.8	29.9	37.9
Sr	Funazoko	saline	−23	13.1	9.2	6.0	28 Jan. 2017	22629	7.34	−3.5	0.47	0.049	146	29.0	34.2

GPS data (Interactive Map file .kmz) are provided online (https://doi.org/10.1016/j.watres.2019.114901).
ND = not determined.
Area name; B = Breivågnipa, L = Langhovde, R = Rundvågshetta, Sl = Skallen, Sr = Skarvsnes.
EC, electrical conductivity at 25°C; Temp, water temperature; $SUVA_{254}$, specific UV absorbance at 254 nm; $S_{275–295}$, spectral slope determined between 275 and 295 nm; DOC, dissolved organic carbon; %HPO, percent hydrophobic fraction of DOC determined by DAX-8 absorption; SPE-DOC recovery; percent solid-phase extracted DOC by PPL resin.

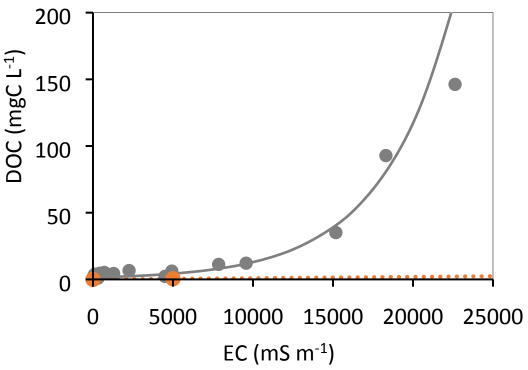

Figure S1. Relationship between electrical conductivity and dissolved organic carbon concentrations. The dotted line represents a hypothetical conservative line when seawater is concentrated, while the solid line fitted for samples. A hypothetical seawater with EC of 5000 mS m^{-1} and DOC of 0.48 mgC L^{-1} (40 µM) was assumed.

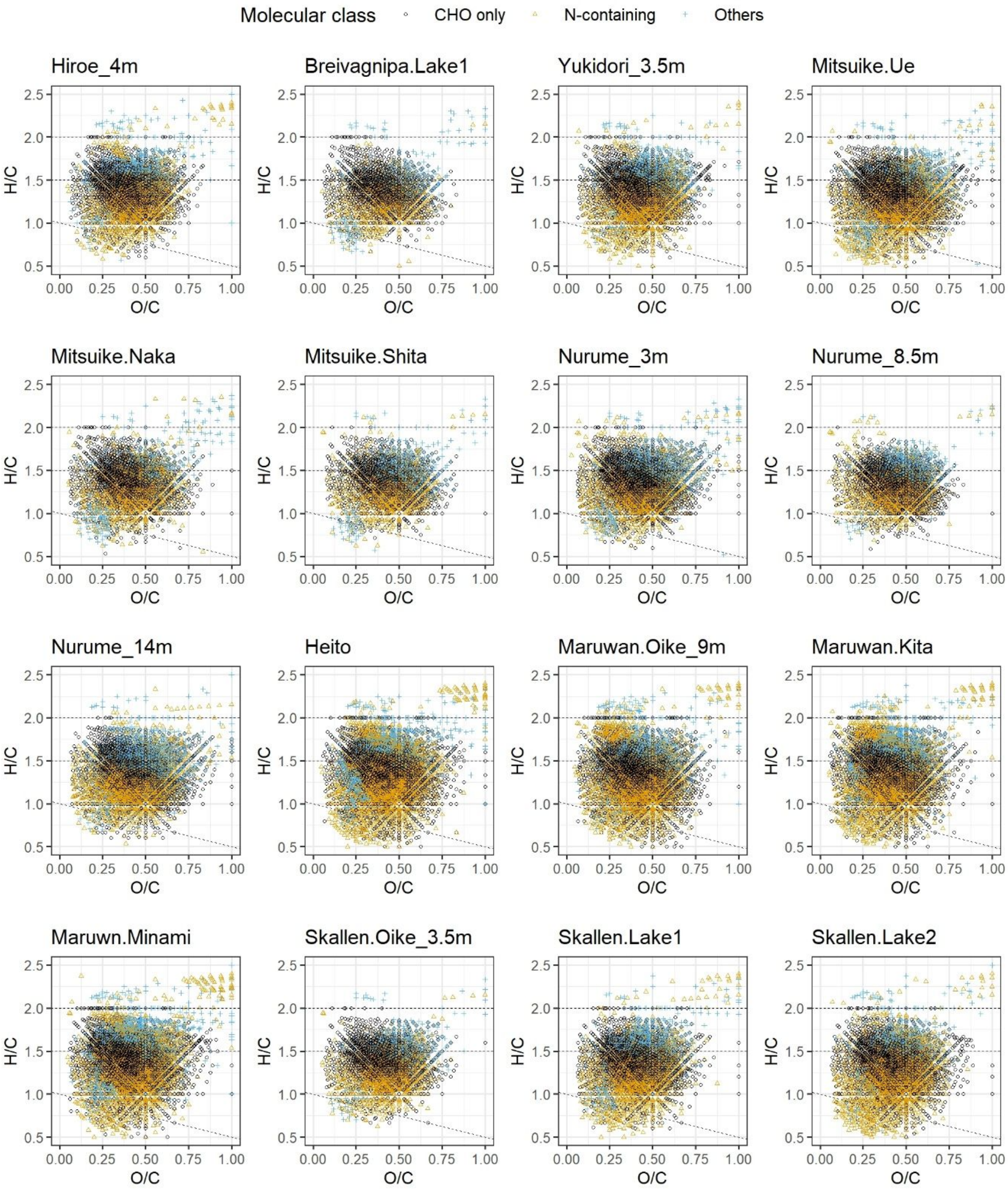

Figure S2. Van Krevelen diagrams of SPE-DOM of all of the studied lakes. The color and shape indicate molecular class, where a molecular formula with only CHO is represented by a black circle, an N-containing molecular formula is by an orange triangle, and an S and/or P-containing (but without N) molecular formula is by a blue cross mark. The dotted lines represent the boundaries for molecular compound groups defined in the main manuscript.

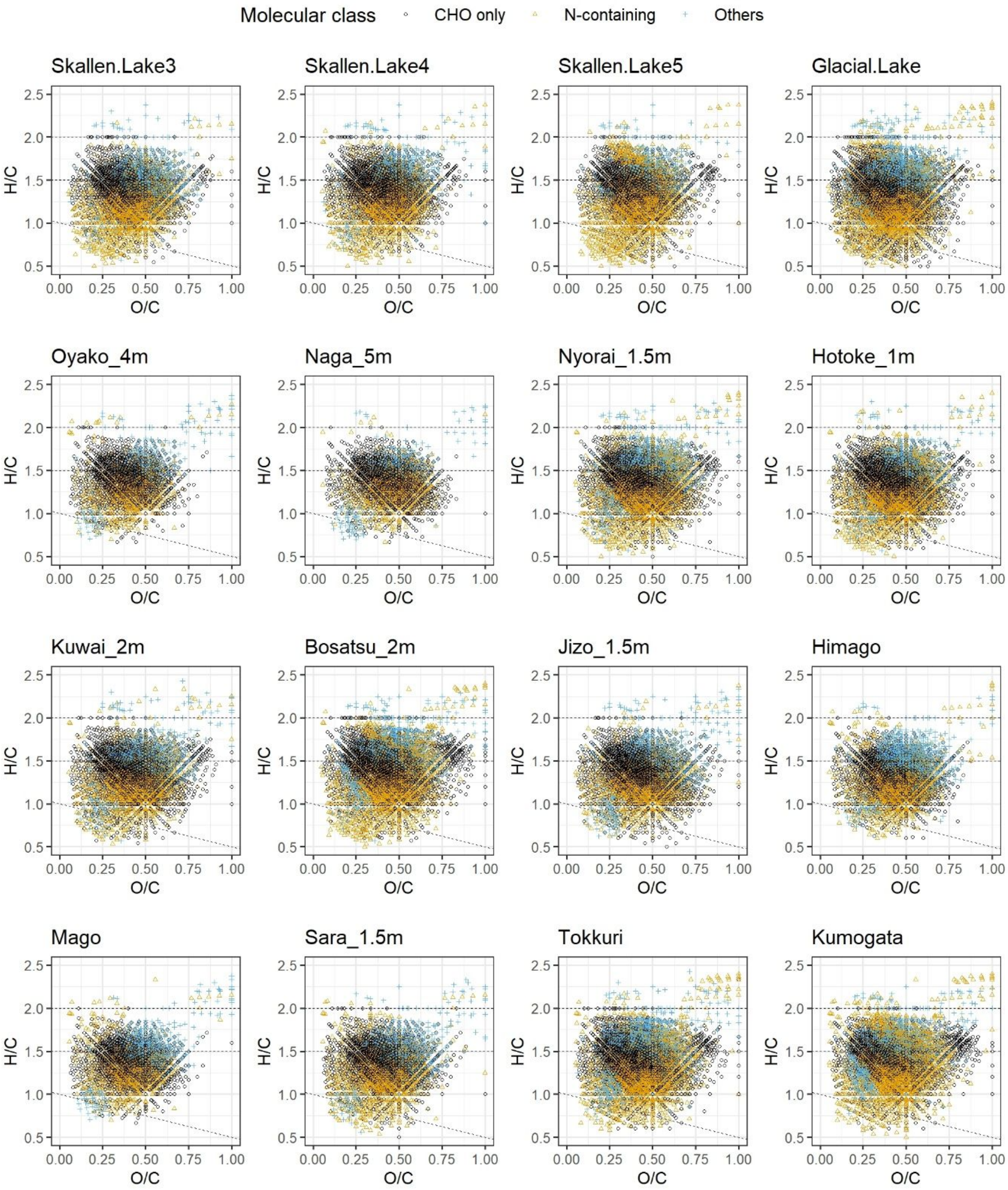

Figure S2. Continued.

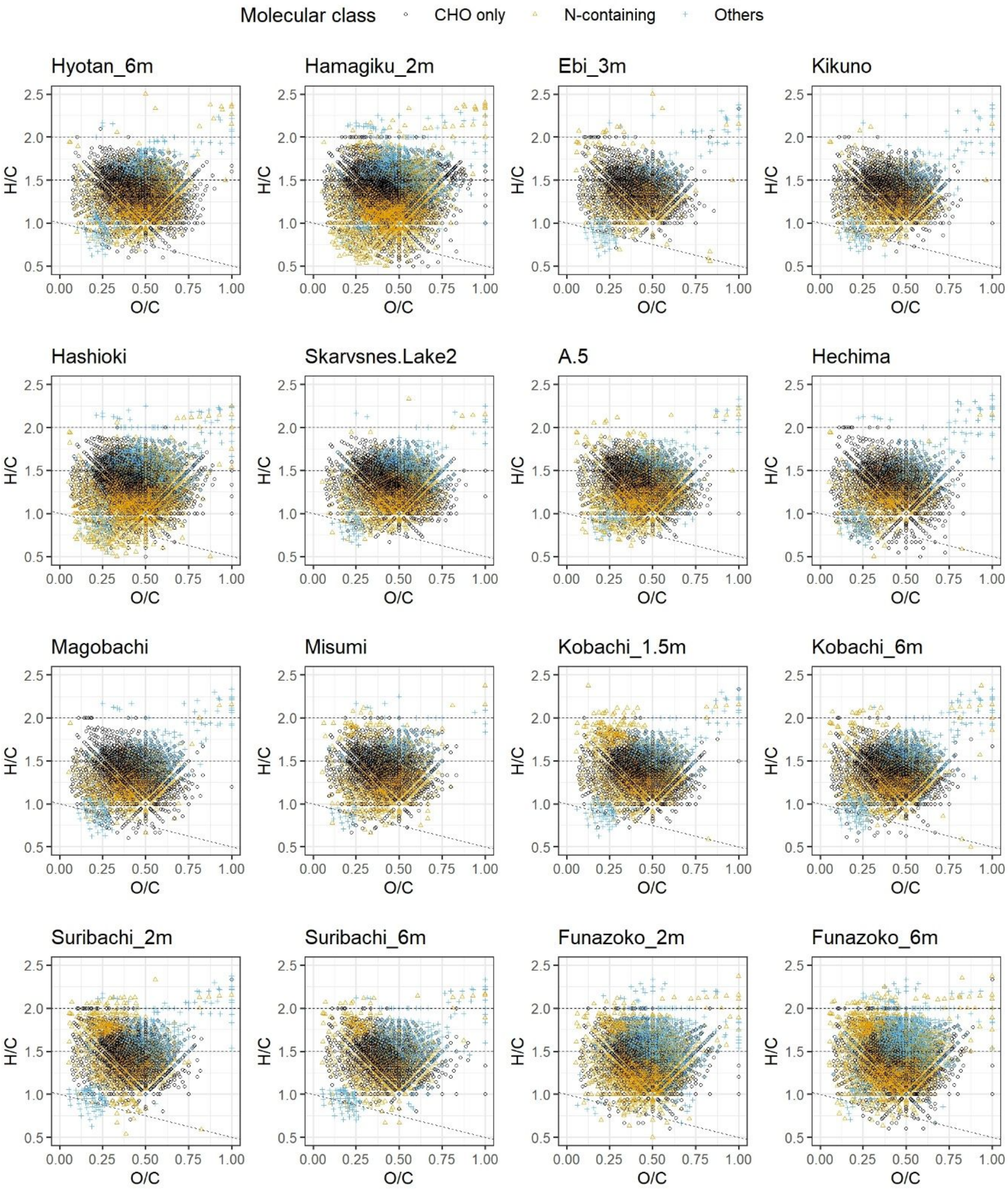

Figure S2. Continued.

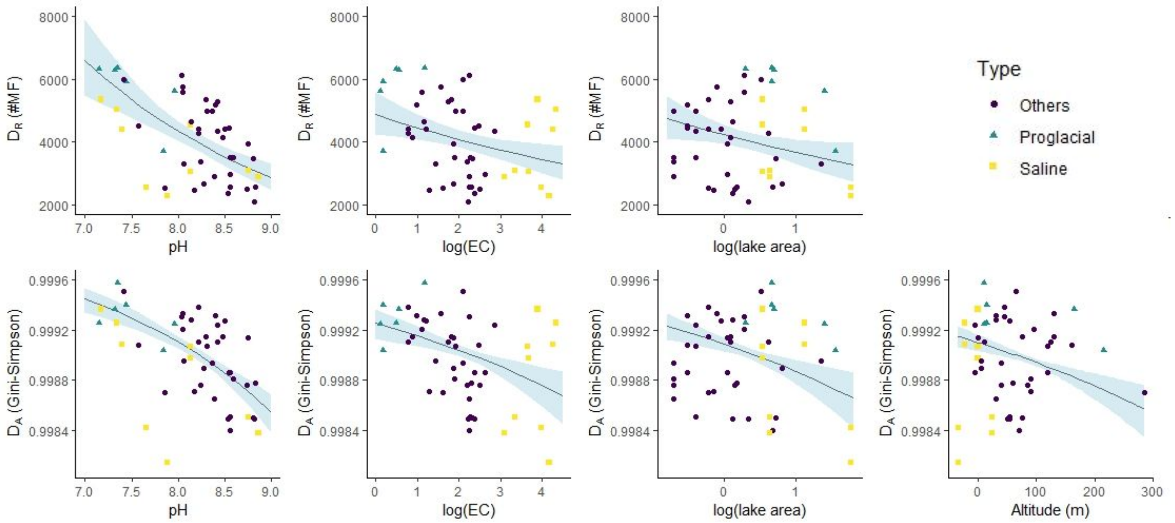

Figure S3. Estimated partial effects of BIC-selected environmental variables on alpha-diversities of DOM analyzed by FT-ICR-MS. Raw data points are overlaid and colored according to the lake type. A negative binomial distribution and beta distribution were assumed for D_R and D_A, respectively. The blue shaded areas indicate the 95% confidence intervals. #MF = number of molecular formulae. Gini-Simpson = abundance-based Gini-Simpson index (Simpson index of diversity).

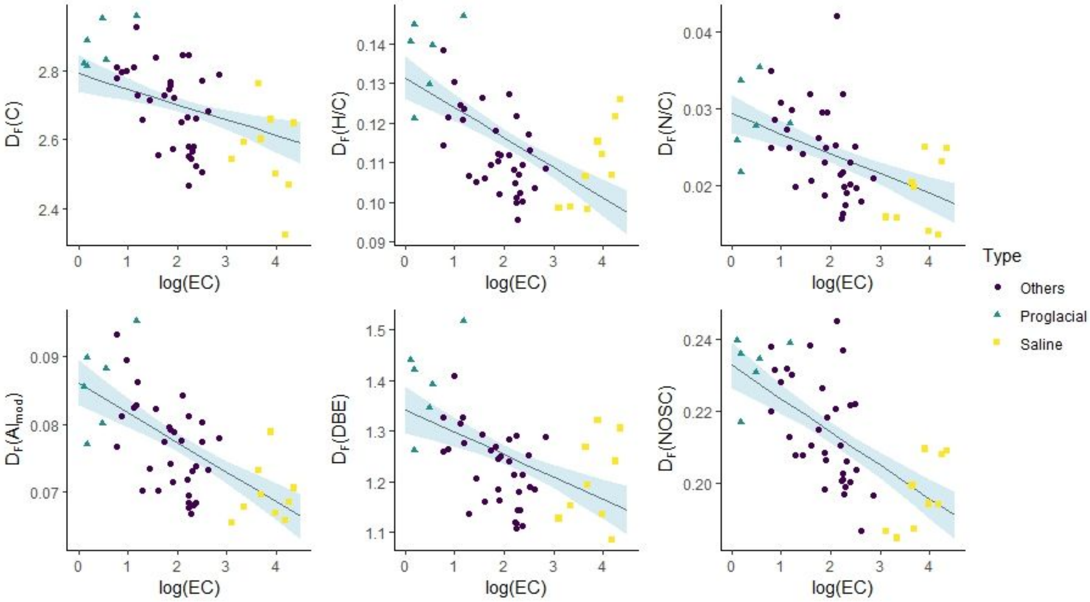

Figure S4. Partial effects of electrical conductivity (EC, log-scale) on functional diversity measures of DOM analyzed by FT-ICR-MS. The blue shaded areas indicate the 95% confidence intervals of the partial effects of EC on functional diversity as estimated via a non-parametric median regression. Raw data points are overlaid and colored according to the lake type.

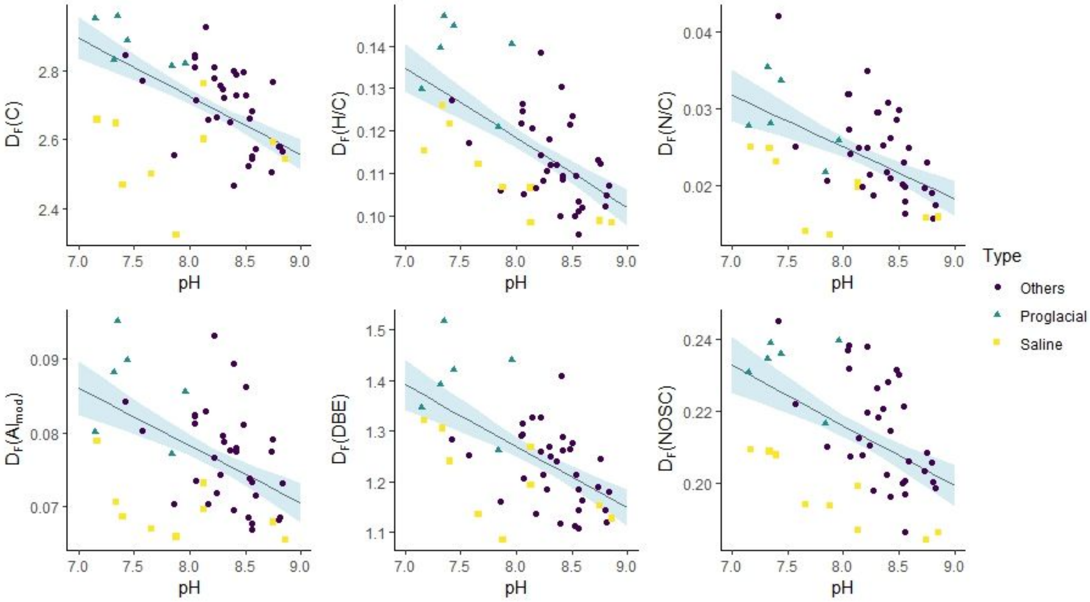

Figure S5. Partial effects of pH on functional diversity measures of DOM analyzed by FT-ICR-MS. The blue shaded areas indicate the 95% confidence intervals of the partial effects of pH on functional diversity as estimated via a non-parametric median regression. Raw data points are overlaid and colored according to the lake type.

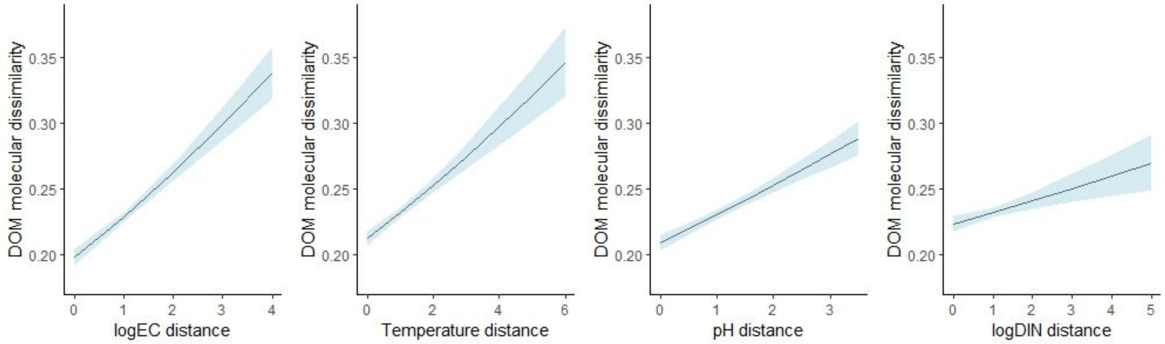

Figure S6. **The beta regression results showing estimated partial effects of dissimilarity in significant environmental variables on DOM molecular dissimilarity (beta-diversity).** The blue shaded areas indicate the 95% confidence intervals. DOM molecular beta-diversity was computed as Bray-Curtis dissimilarity of 9910 normalized FT-ICR-MS peak intensities. The considered environmental variables include water chemistry (electrical conductivity (EC), pH, and temperature), nutrient abundance (DIN, PO_4^{3-}, and SiO_3^{2-}), spatial distance, lake area, and altitude. Note the x-axis corresponds to the distance of the z-scored environmental variable.

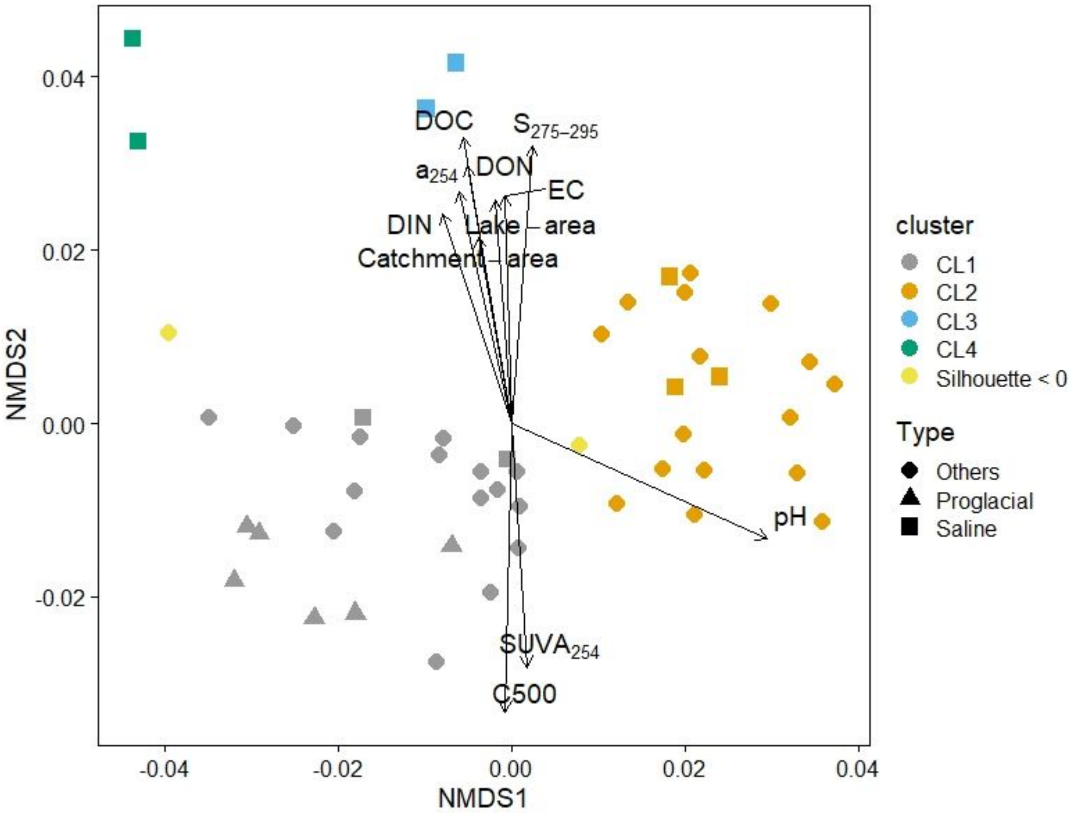

Figure S7. Multivariate analysis of dissolved organic matter molecular data using non-metric multidimensional scaling (stress = 0.0748). Ordinations are based on Jensen-Shannon divergence of DBE_{AI}. The environmental variables and bulk dissolved organic matter properties (arrows) were fitted to the ordination ($P < 0.01$). a_{254}, absorption coefficient of dissolved organic matter at 254 nm; $SUVA_{254}$, DOC-specific ultraviolet absorbance at 254 nm; $S_{275–295}$, spectral slope determined between 275 and 295 nm; C500, the relative abundance (in %) of a humic-like fluorescence component with the emission maximum at 500 nm identified by parallel factor analysis.

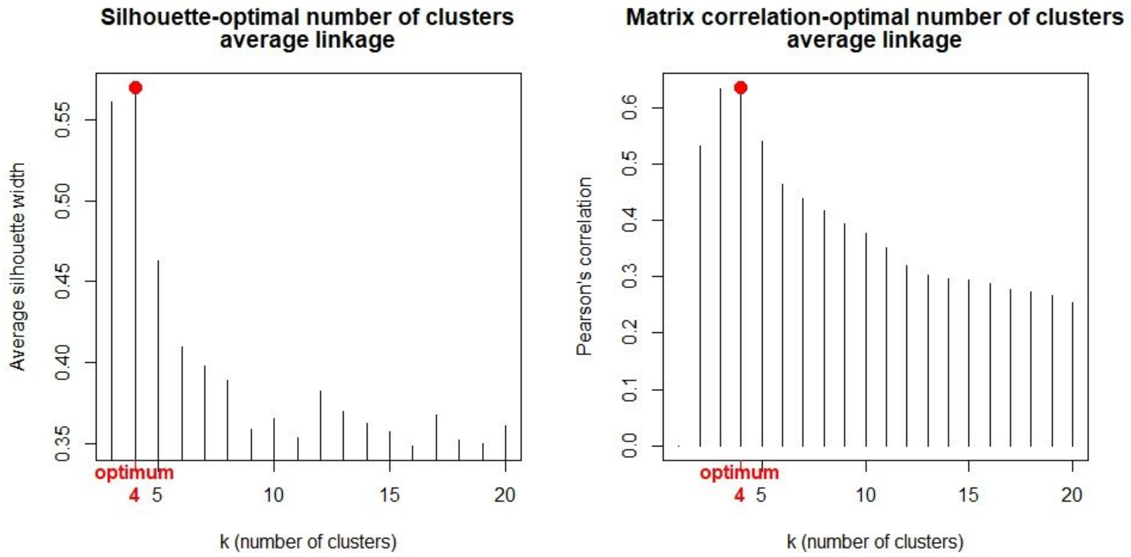

Figure S8. An optimal number of clusters was decided based on silhouette widths and comparison between the dissimilarity matrix and binary matrices representing group allocations. Hierarchical clustering was by average linkage agglomerative clustering of the dissimilarities.

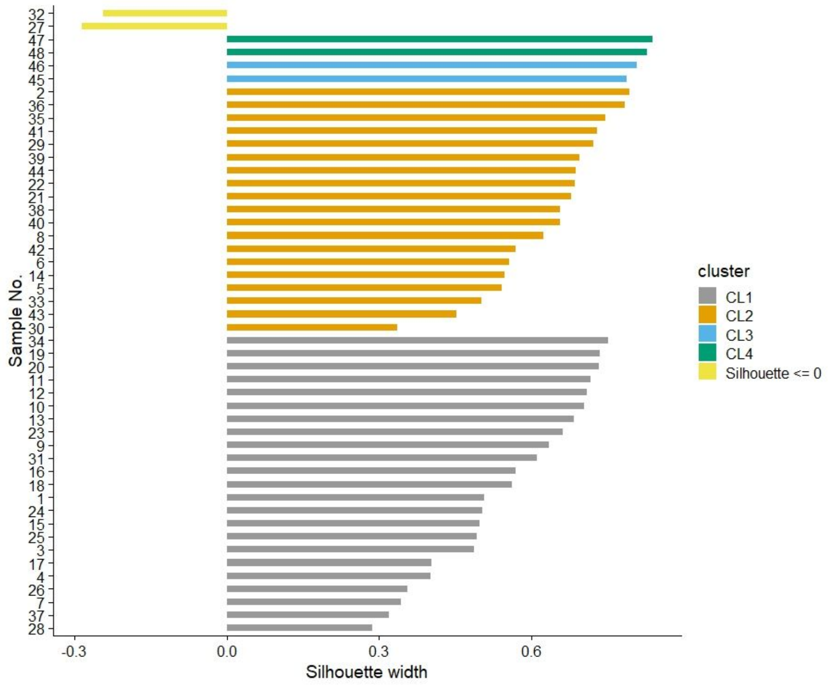

Figure S9. Silhouette widths. Silhouette widths range from -1 to 1 for every molecular formula. Negative values suggest a poor allocation to a cluster (Fig. S5), and such samples were not considered in the further analysis. No 32 = Lake Kumogata, No 27 = Lake Jizo.

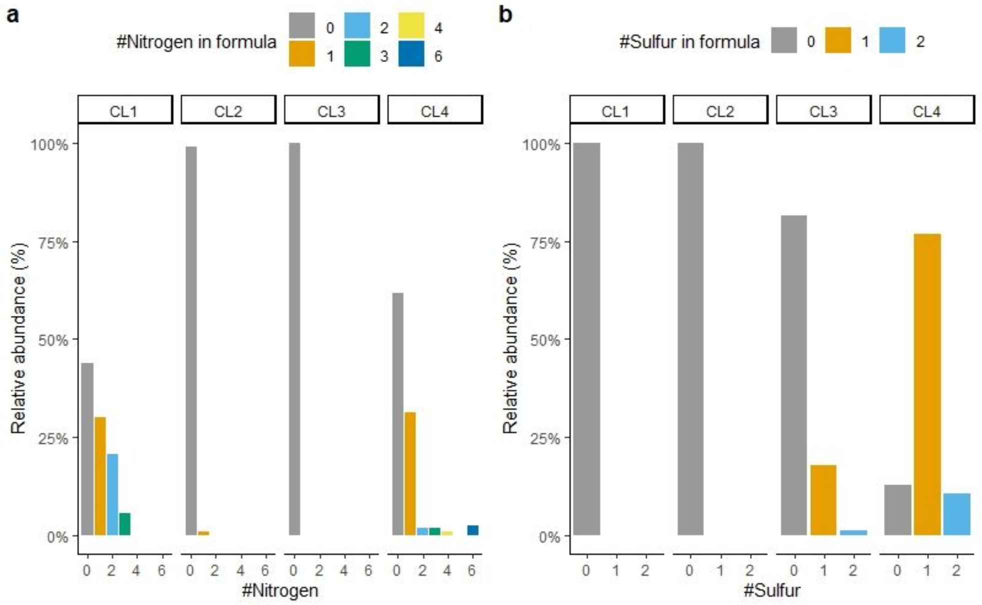

Figure S10. Relative abundance of S or N containing molecular formula for indicator species of each cluster.

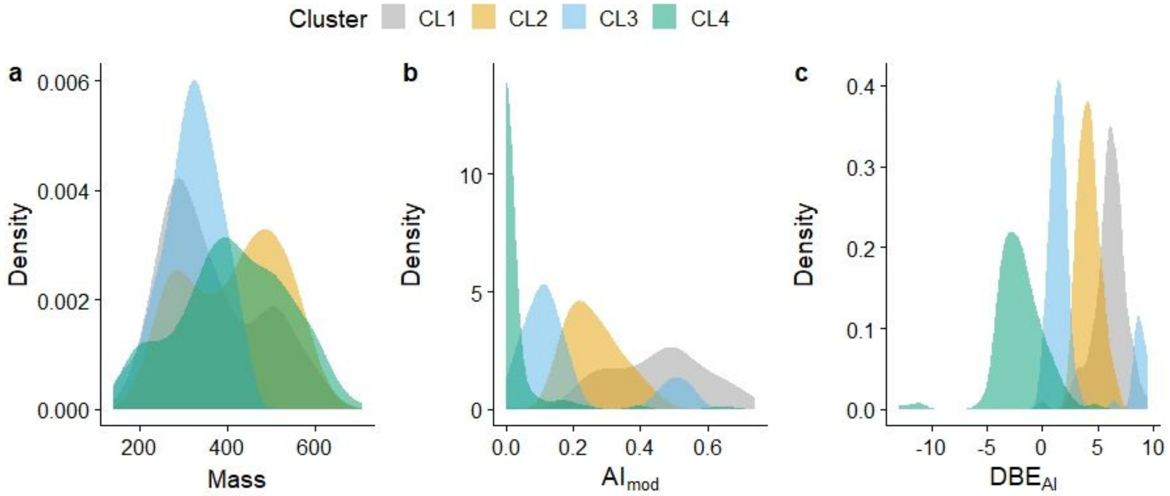

Figure S11. Distributions of mass, AI_{mod}, and DBE_{AI} for indicator species of each cluster.

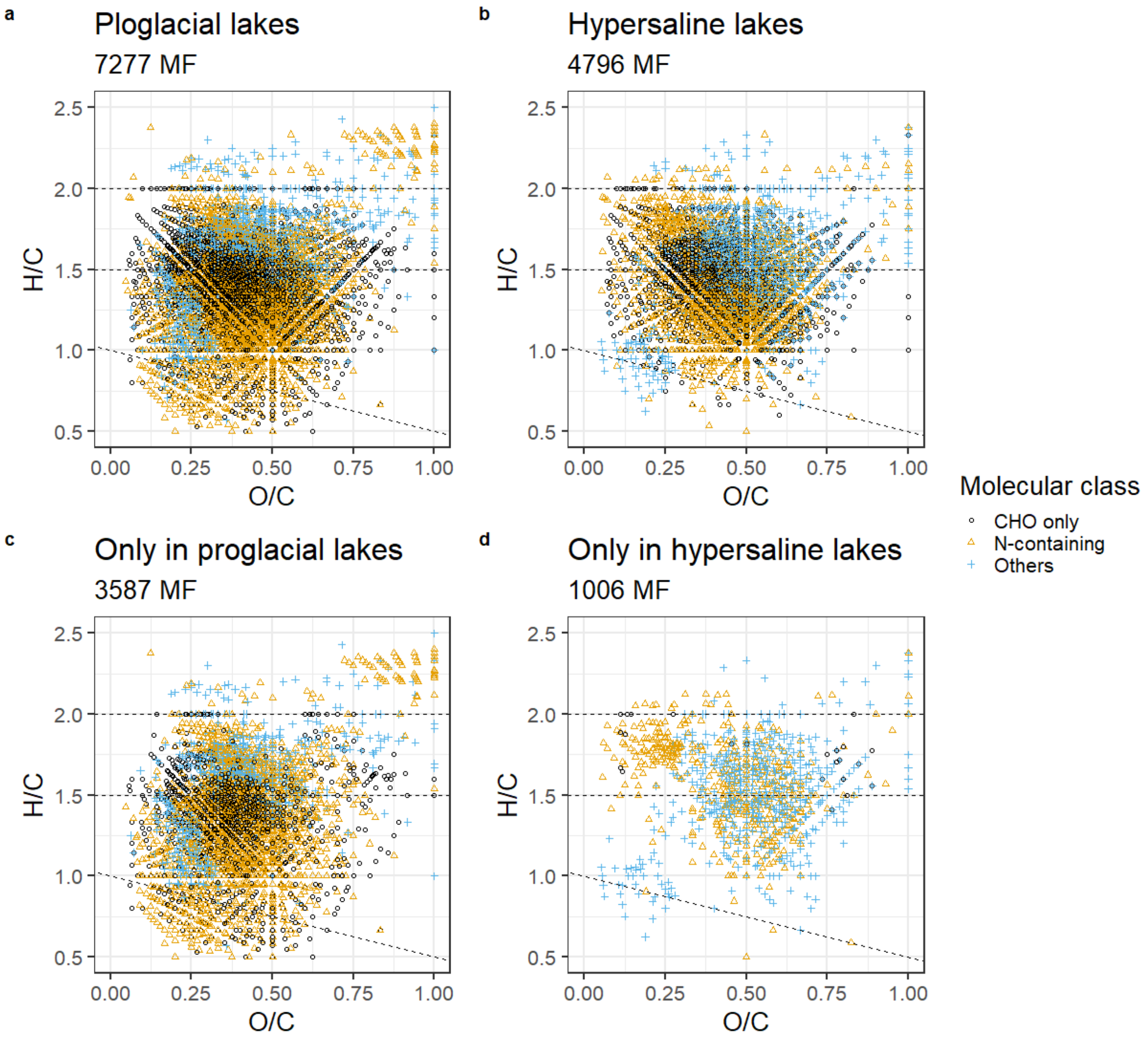

Figure S12. Synthetic van Krevelen diagrams of SPE-DOM of proglacial lakes and hypersaline lakes and their differences. In the upper panel, molecular formulae (MF) detected in at least one of three proglacial lakes (Hiroe_4m, Heito, and Maruwan Minami) (a) or in two hypersaline lakes (Suribachi_2m and Funazoko_2m) (b) are shown. In the lower panel, MF uniquely detected in proglacial lakes (c) or hypersaline lakes (d) are highlighted. The numbers below the title indicate the number of MF detected in each panel. The color and shape indicate molecular class, where a MF with only CHO is represented by a black circle, an N-containing MF is by an orange triangle, and an S and/or P-containing (but without N) MF is by a blue cross mark. The dotted lines represent the boundaries for molecular compound groups defined in the main manuscript.

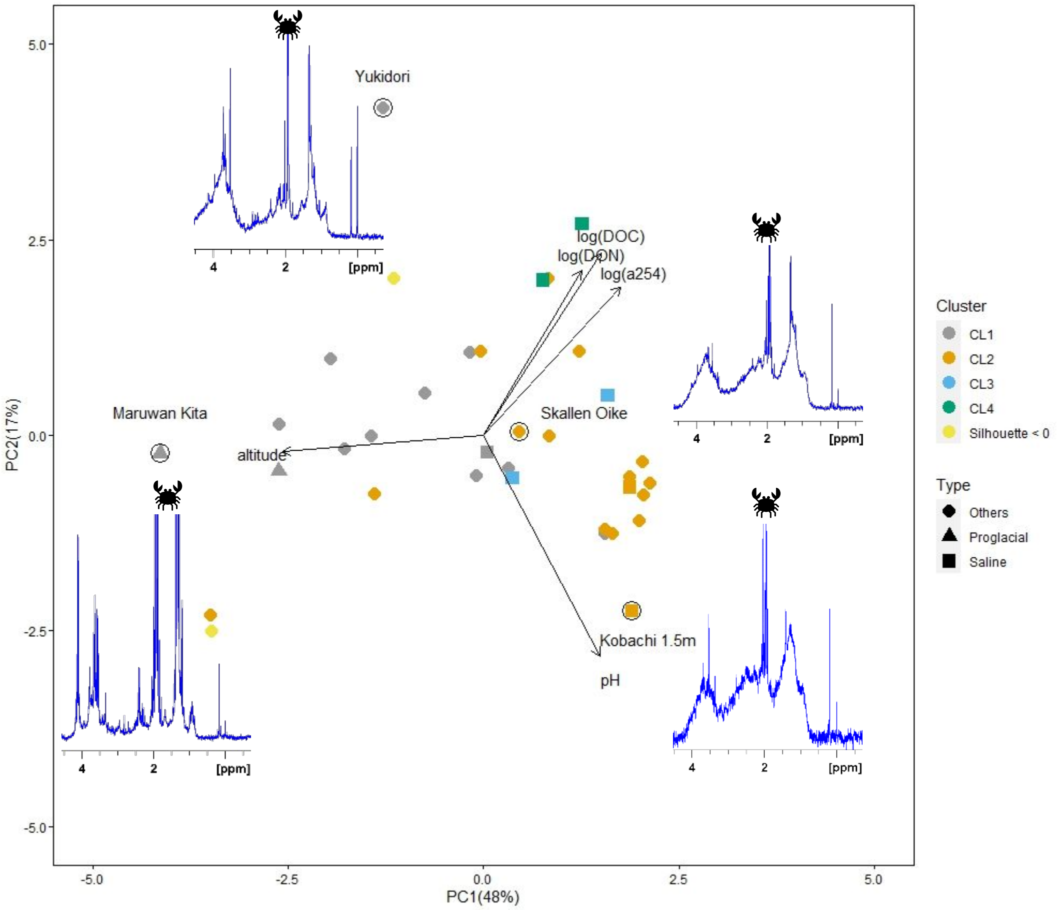

Figure S13. Principal component analysis of bulk water ^{1}H NMR signal intensities. Clustering was according to that identified by NMDS on FT-ICR-MS data of SPE-DOM (Fig. S5). NMR spectra of the four DOM samples marked with the circles are provided to show representative spectra of distinct samples (Yukidori, high PC2 score, carbohydrate rich; Maruwan Kita, low PC1 score, dominated by low-molecular-weight molecules, and Kobachi_1.5 m, high PC1 score & low PC2 score, aliphatic- or functionalized aliphatic-rich lacking sharp signals) and an average sample (Skallen Oike). The chemical shit region of 0.5–4.4 ppm was used for PCA. Signals by CH_3 of lactic and acetic acids are cropped at the crab marks. Samples located between these samples on the PCA ordination space would have NMR spectra intermediate of them. The environmental variables and bulk dissolved organic matter properties (arrows) were fitted to the ordination ($P < 0.05$). a_{254}, absorption coefficient of dissolved organic matter at 254 nm; $SUVA_{254}$, DOC-specific ultraviolet absorbance at 254 nm.
