# Supplementary material for: Determinants of Microbial-Derived Dissolved Organic Matter Diversity in Antarctic Lakes
Source: Environ Sci Technol. 2023 Mar 22;57(13):5464–73. doi: 10.1021/acs.est.3c00249 (PMC10077579; doi:10.1021/acs.est.3c00249)
Supplement: Supplementary file 2 — es3c00249_si_002.pdf [file es3c00249_si_002.pdf]

Cluster	IndVal	Formula	m/z	O/C	H/C	AImod
CL1	0.87262109	C_16 H_15 O_4 N_1	284.092851	0.25	0.938	0.54
CL1	0.854254524	C_15 H_12 O_3 N_2	267.077505	0.2	0.8	0.65
CL1	0.826086957	C_15 H_9 O_5 N_1	282.040815	0.333	0.6	0.74
CL1	0.826086957	C_17 H_15 O_7 N_1	344.077612	0.412	0.882	0.52
CL1	0.796982303	C_17 H_14 O_6	313.071745	0.353	0.824	0.57
CL1	0.792220346	C_17 H_17 O_4 N_1	298.108499	0.235	1	0.5
CL1	0.789475047	C_16 H_11 O_4 N_1	280.061512	0.25	0.688	0.69
CL1	0.782608696	C_15 H_11 O_4 N_1	268.061556	0.267	0.733	0.67
CL1	0.758932541	C_18 H_14 O_8	357.061625	0.444	0.778	0.57
CL1	0.739130435	C_15 H_10 O_3 N_2	265.06184	0.2	0.667	0.74
CL1	0.739130435	C_15 H_10 O_4 N_2	281.056766	0.267	0.667	0.73
CL1	0.739130435	C_17 H_14 O_5	297.076828	0.294	0.824	0.59
CL1	0.735420265	C_14 H_11 O_3 N_1	240.066614	0.214	0.786	0.65
CL1	0.733202189	C_15 H_11 O_6 N_1	300.051341	0.4	0.733	0.64
CL1	0.729589147	C_14 H_12 O_3	227.07135	0.214	0.857	0.6
CL1	0.72850383	C_16 H_12 O_4 N_2	295.072465	0.25	0.75	0.67
CL1	0.722496796	C_17 H_13 O_3 N_1	278.082286	0.176	0.765	0.66
CL1	0.713238371	C_26 H_31 O_9 N_3	528.198797	0.346	1.192	0.3
CL1	0.695652174	C_24 H_25 O_8 N_3	482.156943	0.333	1.042	0.41
CL1	0.695652174	C_23 H_23 O_10 N_3	500.131122	0.435	1	0.4
CL1	0.695652174	C_26 H_31 O_8 N_3	512.203869	0.308	1.192	0.32
CL1	0.695652174	C_29 H_34 O_12	573.197746	0.414	1.172	0.3
CL1	0.687384811	C_19 H_18 O_6	341.103065	0.316	0.947	0.5
CL1	0.671483029	C_22 H_23 O_10 N_1	460.124918	0.455	1.045	0.38
CL1	0.666388399	C_15 H_12 O_4 N_2	283.072445	0.267	0.8	0.64
CL1	0.659288656	C_17 H_32 O_4	299.222773	0.235	1.882	0
CL1	0.658673491	C_16 H_17 O_3 N_1	270.113554	0.188	1.062	0.48
CL1	0.650347344	C_16 H_13 O_7 N_1	330.061945	0.438	0.812	0.57
CL1	0.640004303	C_19 H_19 O_9 N_1	404.098728	0.474	1	0.41
CL1	0.639305054	C_17 H_15 O_8 N_1	360.072465	0.471	0.882	0.5
CL1	0.637521446	C_19 H_19 O_7 N_1	372.108887	0.368	1	0.45
CL1	0.619378053	C_11 H_8 O_4 N_2	231.041149	0.364	0.727	0.71
CL1	0.618835639	C_25 H_27 O_10 N_3	528.162442	0.4	1.08	0.35
CL1	0.611576225	C_24 H_27 O_12 N_1	520.146086	0.5	1.125	0.29
CL1	0.601641168	C_20 H_20 O_7	371.113602	0.35	1	0.45
CL1	0.594178815	C_16 H_14 O_7	317.0667	0.438	0.875	0.52
CL1	0.588821398	C_17 H_16 O_9 N_2	391.07834	0.529	0.941	0.43
CL1	0.587216584	C_14 H_10 O_6	273.040441	0.429	0.714	0.64

CL1	0.562152862	C_14 H_11 O_6 N_1	288.05134	0.429	0.786	0.6
CL1	0.560545085	C_31 H_40 O_12	603.244705	0.387	1.29	0.24
CL1	0.558277262	C_21 H_22 O_7	385.129273	0.333	1.048	0.43
CL1	0.542178748	C_21 H_22 O_8 N_2	429.130382	0.381	1.048	0.4
CL1	0.537065708	C_21 H_23 O_8 N_1	416.135066	0.381	1.095	0.38
CL1	0.531648415	C_17 H_16 O_8 N_2	375.083361	0.471	0.941	0.45
CL1	0.528699882	C_16 H_14 O_8	333.061611	0.5	0.875	0.5
CL1	0.520553215	C_20 H_20 O_9 N_2	431.109642	0.45	1	0.41
CL1	0.519814842	C_15 H_12 O_6	287.056132	0.4	0.8	0.58
CL1	0.51356329	C_16 H_14 O_5	285.076861	0.312	0.875	0.56
CL1	0.511291543	C_21 H_21 O_10 N_3	474.115475	0.476	1	0.38
CL1	0.510502962	C_15 H_13 O_8 N_1	334.056821	0.533	0.867	0.5
CL1	0.509469242	C_15 H_14 O_4 N_2	285.088058	0.267	0.933	0.55
CL1	0.508769844	C_25 H_28 O_10	487.16101	0.4	1.12	0.35
CL1	0.50848429	C_20 H_21 O_8 N_1	402.119415	0.4	1.05	0.4
CL1	0.502884417	C_31 H_40 O_13	619.239621	0.419	1.29	0.22
CL1	0.496577665	C_15 H_15 O_3 N_1	256.097938	0.2	1	0.52
CL1	0.478674123	C_16 H_16 O_4 N_2	299.103717	0.25	1	0.5
CL1	0.478389312	C_30 H_38 O_13	605.224005	0.433	1.267	0.23
CL1	0.47743981	C_13 H_10 O_4	229.050646	0.308	0.769	0.64
CL1	0.473347529	C_15 H_13 O_7 N_1	318.061902	0.467	0.867	0.52
CL1	0.472479669	C_30 H_38 O_11	573.234221	0.367	1.267	0.27
CL1	0.471281463	C_12 H_10 O_5 N_2	261.051675	0.417	0.833	0.6
CL1	0.470263892	C_13 H_11 O_3 N_1	228.066626	0.231	0.846	0.62
CL1	0.465539775	C_15 H_13 O_6 N_1	302.067009	0.4	0.867	0.55
CL1	0.458186008	C_26 H_32 O_8	471.202474	0.308	1.231	0.32
CL1	0.45218286	C_16 H_15 O_8 N_1	348.072508	0.5	0.938	0.45
CL1	0.442237257	C_11 H_10 O_3 N_2	217.061852	0.273	0.909	0.6
CL1	0.442126315	C_25 H_28 O_11	503.155922	0.44	1.12	0.33
CL1	0.441375019	C_14 H_12 O_4 N_2	271.072411	0.286	0.857	0.6
CL1	0.439082174	C_27 H_32 O_10	515.192244	0.37	1.185	0.32
CL1	0.425848846	C_16 H_14 O_6	301.071744	0.375	0.875	0.54
CL1	0.424924891	C_18 H_22 O_5 N_2	345.145601	0.278	1.222	0.33
CL1	0.410815254	C_24 H_32 O_6	415.212577	0.25	1.333	0.29
CL1	0.403829816	C_21 H_24 O_6	371.150033	0.286	1.143	0.39
CL1	0.40159732	C_17 H_16 O_7	331.082352	0.412	0.941	0.48
CL1	0.400819179	C_14 H_14 O_3 N_2	257.093184	0.214	1	0.52
CL1	0.395459082	C_22 H_26 O_6	385.165659	0.273	1.182	0.37
CL1	0.39356097	C_13 H_12 O_6 N_2	291.062264	0.462	0.923	0.5

CL1	0.392695095	C_28 H_40 O_7	487.270163	0.25	1.429	0.22
CL1	0.392599353	C_17 H_16 O_6	315.087417	0.353	0.941	0.5
CL1	0.384606468	C_22 H_30 O_5	373.202056	0.227	1.364	0.28
CL1	0.382964871	C_25 H_36 O_6	431.243927	0.24	1.44	0.23
CL1	0.379923251	C_13 H_11 O_4 N_1	244.061544	0.308	0.846	0.6
CL1	0.377147898	C_11 H_10 O_4 N_2	233.056797	0.364	0.909	0.57
CL1	0.376032271	C_15 H_14 O_4	257.081915	0.267	0.933	0.54
CL1	0.37256173	C_30 H_40 O_11	575.249829	0.367	1.333	0.22
CL1	0.369517251	C_27 H_38 O_7	473.254443	0.259	1.407	0.23
CL1	0.366857998	C_26 H_30 O_12	533.166427	0.462	1.154	0.3
CL1	0.365850856	C_28 H_36 O_9	515.228654	0.321	1.286	0.28
CL1	0.36240333	C_14 H_13 O_4 N_1	258.077198	0.286	0.929	0.55
CL1	0.361675395	C_28 H_38 O_8	501.249403	0.286	1.357	0.25
CL1	0.35922393	C_15 H_14 O_5	273.076865	0.333	0.933	0.52
CL1	0.353533392	C_17 H_17 O_7 N_1	346.093209	0.412	1	0.44
CL1	0.353350135	C_23 H_28 O_7	415.176247	0.304	1.217	0.33
CL1	0.342554065	C_18 H_18 O_7	345.097986	0.389	1	0.45
CL1	0.342533299	C_12 H_10 O_4	217.050624	0.333	0.833	0.6
CL1	0.340062971	C_18 H_18 O_6	329.103055	0.333	1	0.47
CL1	0.339154564	C_14 H_14 O_7 N_2	321.072824	0.5	1	0.41
CL1	0.339079825	C_28 H_36 O_10	531.223552	0.357	1.286	0.26
CL1	0.334186313	C_29 H_40 O_9	531.259961	0.31	1.379	0.22
CL1	0.331947252	C_17 H_18 O_9 N_2	393.093927	0.529	1.059	0.33
CL1	0.33041397	C_29 H_38 O_13	593.223949	0.448	1.31	0.2
CL1	0.325463502	C_12 H_12 O_5 N_2	263.067324	0.417	1	0.47
CL1	0.321974346	C_26 H_32 O_9	487.197342	0.346	1.231	0.3
CL1	0.319483496	C_16 H_16 O_4	271.097568	0.25	1	0.5
CL1	0.318289575	C_9 H_9 O_4 N_1	194.045872	0.444	1	0.5
CL1	0.317615091	C_16 H_16 O_5	287.092496	0.312	1	0.48
CL1	0.311035813	C_11 H_11 O_5 N_1	236.05644	0.455	1	0.47
CL1	0.305349373	C_11 H_11 O_6 N_1	252.051347	0.545	1	0.43
CL1	0.290483662	C_16 H_18 O_9 N_2	381.093968	0.562	1.125	0.26
CL1	0.287985993	C_16 H_16 O_6	303.087393	0.375	1	0.46
CL1	0.284965186	C_28 H_36 O_11	547.218515	0.393	1.286	0.24
CL1	0.266751353	C_17 H_18 O_7	333.097975	0.412	1.059	0.41
CL1	0.263585787	C_10 H_11 O_4 N_1	208.061538	0.4	1.1	0.43
CL2	0.369908817	C_24 H_28 O_8	443.171116	0.333	1.167	0.35
CL2	0.364371971	C_26 H_34 O_8	473.218106	0.308	1.308	0.27
CL2	0.352515743	C_17 H_20 O_3	271.133988	0.176	1.176	0.42

CL2	0.338485021	C_25 H_34 O_7	445.223176	0.28	1.36	0.26
CL2	0.337184596	C_15 H_16 O_3	243.102654	0.2	1.067	0.48
CL2	0.335325786	C_19 H_22 O_5	329.139455	0.263	1.158	0.39
CL2	0.332224065	C_16 H_20 O_3	259.133978	0.188	1.25	0.38
CL2	0.326523512	C_28 H_38 O_9	517.244339	0.321	1.357	0.23
CL2	0.323926911	C_29 H_38 O_11	561.234181	0.379	1.31	0.23
CL2	0.322042816	C_24 H_32 O_7	431.207524	0.292	1.333	0.27
CL2	0.32041686	C_29 H_42 O_9	533.275648	0.31	1.448	0.18
CL2	0.316918738	C_15 H_16 O_4	259.097601	0.267	1.067	0.46
CL2	0.313922439	C_26 H_34 O_14	569.187579	0.538	1.308	0.16
CL2	0.313203699	C_29 H_38 O_12	577.229082	0.414	1.31	0.22
CL2	0.310922011	C_27 H_34 O_10	517.207955	0.37	1.259	0.27
CL2	0.308736308	C_30 H_44 O_11	579.281114	0.367	1.467	0.14
CL2	0.307335446	C_25 H_30 O_9	473.181722	0.36	1.2	0.32
CL2	0.298959988	C_15 H_18 O_4	261.113227	0.267	1.2	0.38
CL2	0.296784114	C_14 H_18 O_4	249.11325	0.286	1.286	0.33
CL2	0.295950958	C_14 H_16 O_4	247.097597	0.286	1.143	0.42
CL2	0.295237665	C_20 H_23 O_9 N_1	420.130005	0.45	1.15	0.31
CL2	0.295230251	C_16 H_20 O_5	291.123811	0.312	1.25	0.33
CL2	0.294208241	C_14 H_16 O_7	295.082311	0.5	1.143	0.33
CL2	0.293028033	C_29 H_40 O_11	563.249804	0.379	1.379	0.19
CL2	0.292991268	C_30 H_40 O_13	607.2396	0.433	1.333	0.19
CL2	0.292497281	C_26 H_36 O_14	571.203265	0.538	1.385	0.11
CL2	0.288996167	C_15 H_18 O_5	277.108132	0.333	1.2	0.36
CL2	0.288776197	C_15 H_20 O_5	279.123818	0.333	1.333	0.28
CL2	0.287181096	C_12 H_14 O_3	205.087012	0.25	1.167	0.43
CL2	0.286182653	C_15 H_18 O_6	293.103055	0.4	1.2	0.33
CL2	0.285914457	C_13 H_14 O_6	265.071778	0.462	1.077	0.4
CL2	0.285824093	C_16 H_20 O_6	307.118729	0.375	1.25	0.31
CL2	0.285450159	C_14 H_16 O_5	263.092487	0.357	1.143	0.39
CL2	0.285149244	C_30 H_44 O_12	595.276041	0.4	1.467	0.12
CL2	0.284748611	C_25 H_34 O_9	477.213001	0.36	1.36	0.22
CL2	0.284448156	C_14 H_16 O_6	279.087392	0.429	1.143	0.36
CL2	0.284380625	C_25 H_32 O_9	475.197375	0.36	1.28	0.27
CL2	0.284287048	C_17 H_20 O_6	319.11872	0.353	1.176	0.36
CL2	0.284222265	C_16 H_18 O_5	289.108151	0.312	1.125	0.41
CL2	0.28285158	C_23 H_30 O_10	465.176655	0.435	1.304	0.22
CL2	0.282393512	C_26 H_34 O_11	521.202874	0.423	1.308	0.22
CL2	0.281547567	C_19 H_24 O_9	395.134766	0.474	1.263	0.24

CL2	0.280987866	C_24 H_32 O_10	479.192307	0.417	1.333	0.21
CL2	0.280841273	C_13 H_16 O_4	235.097597	0.308	1.231	0.36
CL2	0.2807422	C_24 H_32 O_9	463.197364	0.375	1.333	0.23
CL2	0.280627474	C_17 H_20 O_8	351.108547	0.471	1.176	0.31
CL2	0.280611208	C_27 H_36 O_10	519.223595	0.37	1.333	0.23
CL2	0.280596646	C_29 H_40 O_13	595.239616	0.448	1.379	0.16
CL2	0.279946357	C_21 H_26 O_7	389.160578	0.333	1.238	0.31
CL2	0.279937738	C_23 H_30 O_11	481.171525	0.478	1.304	0.2
CL2	0.279560909	C_24 H_30 O_9	461.181737	0.375	1.25	0.28
CL2	0.279526096	C_15 H_18 O_7	309.097953	0.467	1.2	0.3
CL2	0.279007112	C_18 H_22 O_7	349.129294	0.389	1.222	0.31
CL2	0.278441852	C_15 H_16 O_5	275.092514	0.333	1.067	0.44
CL2	0.278199037	C_13 H_14 O_5	249.076865	0.385	1.077	0.43
CL2	0.277910564	C_30 H_44 O_13	611.270924	0.433	1.467	0.11
CL2	0.277891307	C_16 H_20 O_7	323.11361	0.438	1.25	0.28
CL2	0.277170711	C_20 H_26 O_9	409.150425	0.45	1.3	0.23
CL2	0.277077197	C_13 H_16 O_5	251.092479	0.385	1.231	0.33
CL2	0.276414917	C_23 H_28 O_9	447.166062	0.391	1.217	0.3
CL2	0.276048573	C_26 H_34 O_10	505.207905	0.385	1.308	0.24
CL2	0.275982036	C_14 H_18 O_5	265.108131	0.357	1.286	0.3
CL2	0.275792522	C_26 H_36 O_10	507.223584	0.385	1.385	0.19
CL2	0.275400961	C_14 H_18 O_6	281.103046	0.429	1.286	0.27
CL2	0.274713463	C_26 H_36 O_11	523.218518	0.423	1.385	0.17
CL2	0.274587691	C_15 H_16 O_7	307.082347	0.467	1.067	0.39
CL2	0.274300972	C_16 H_18 O_7	321.097954	0.438	1.125	0.36
CL2	0.274047118	C_17 H_20 O_7	335.113606	0.412	1.176	0.33
CL2	0.27344854	C_12 H_14 O_5	237.076845	0.417	1.167	0.37
CL2	0.273046638	C_17 H_22 O_7	337.12927	0.412	1.294	0.26
CL2	0.272936451	C_22 H_28 O_10	451.160973	0.455	1.273	0.24
CL2	0.272814835	C_23 H_32 O_10	467.192296	0.435	1.391	0.17
CL2	0.272783669	C_25 H_34 O_10	493.207918	0.4	1.36	0.2
CL2	0.272637486	C_18 H_24 O_7	351.14495	0.389	1.333	0.24
CL2	0.272636559	C_17 H_22 O_6	321.134346	0.353	1.294	0.29
CL2	0.272256263	C_17 H_22 O_8	353.124167	0.471	1.294	0.23
CL2	0.272198068	C_23 H_28 O_8	431.171119	0.348	1.217	0.32
CL2	0.271663948	C_23 H_30 O_9	449.181713	0.391	1.304	0.24
CL2	0.271333333	C_22 H_28 O_9	435.166074	0.409	1.273	0.26
CL2	0.271302578	C_27 H_36 O_11	535.218514	0.407	1.333	0.21
CL2	0.270761609	C_24 H_34 O_9	465.212995	0.375	1.417	0.18

CL2	0.270677129	C_25 H_34 O_12	525.197767	0.48	1.36	0.16
CL2	0.270537681	C_23 H_30 O_8	433.186757	0.348	1.304	0.26
CL2	0.270395817	C_27 H_34 O_12	549.197744	0.444	1.259	0.24
CL2	0.270059052	C_19 H_26 O_9	397.150387	0.474	1.368	0.17
CL2	0.270023408	C_28 H_38 O_12	565.2291	0.429	1.357	0.18
CL2	0.270022255	C_25 H_32 O_10	491.192258	0.4	1.28	0.25
CL2	0.269794994	C_20 H_26 O_7	377.160553	0.35	1.3	0.27
CL2	0.269636975	C_24 H_32 O_11	495.187164	0.458	1.333	0.19
CL2	0.269519673	C_27 H_38 O_10	521.239209	0.37	1.407	0.18
CL2	0.269302318	C_22 H_28 O_8	419.171139	0.364	1.273	0.28
CL2	0.269093739	C_18 H_22 O_8	365.124167	0.444	1.222	0.29
CL2	0.269034919	C_27 H_36 O_12	551.213392	0.444	1.333	0.19
CL2	0.269021134	C_20 H_24 O_8	391.13987	0.4	1.2	0.31
CL2	0.268358121	C_19 H_24 O_8	379.139824	0.421	1.263	0.27
CL2	0.267911437	C_21 H_28 O_9	423.166066	0.429	1.333	0.21
CL2	0.267675911	C_20 H_26 O_8	393.155473	0.4	1.3	0.25
CL2	0.267639127	C_22 H_30 O_10	453.17662	0.455	1.364	0.18
CL2	0.267095153	C_25 H_32 O_11	507.18715	0.44	1.28	0.23
CL2	0.266530504	C_16 H_18 O_6	305.103045	0.375	1.125	0.38
CL2	0.265559295	C_21 H_28 O_8	407.171143	0.381	1.333	0.24
CL2	0.264996815	C_26 H_34 O_12	537.197773	0.462	1.308	0.2
CL2	0.264294812	C_19 H_26 O_8	381.155491	0.421	1.368	0.2
CL2	0.263211936	C_24 H_34 O_10	481.20789	0.417	1.417	0.16
CL2	0.263129557	C_25 H_34 O_11	509.20281	0.44	1.36	0.18
CL2	0.262864325	C_18 H_24 O_8	367.13982	0.444	1.333	0.21
CL2	0.261208893	C_27 H_38 O_12	553.229086	0.444	1.407	0.14
CL2	0.260006446	C_22 H_30 O_9	437.1817	0.409	1.364	0.2
CL2	0.259458572	C_28 H_38 O_11	549.234118	0.393	1.357	0.2
CL2	0.258651034	C_23 H_32 O_9	451.197322	0.391	1.391	0.19
CL2	0.258226425	C_24 H_34 O_11	497.202819	0.458	1.417	0.14
CL2	0.256259641	C_26 H_36 O_12	539.213417	0.462	1.385	0.15
CL2	0.253834055	C_13 H_15 O_6 N_1	280.082666	0.462	1.154	0.33
CL2	0.252960357	C_25 H_36 O_11	511.218454	0.44	1.44	0.13
CL2	0.252394392	C_28 H_40 O_12	567.244676	0.429	1.429	0.14
CL3	1	C_19 H_20 O_1 S_1	295.11633	0.053	1.053	0.49
CL3	1	C_21 H_22 O_2 S_1	337.126865	0.095	1.048	0.47
CL3	1	C_19 H_20 O_3 S_2	359.078218	0.158	1.053	0.42
CL3	0.97496302	C_17 H_16 O_1 S_1	267.084997	0.059	0.941	0.55
CL3	0.973269933	C_12 H_15 O_6 S_1 P_1	317.025511	0.5	1.25	0.14

CL3	0.972674914	C_22 H_22 O_5 S_1	397.111632	0.227	1	0.46
CL3	0.967191748	C_21 H_22 O_3 S_1	353.121777	0.143	1.048	0.46
CL3	0.961960902	C_20 H_20 O_3 S_1	339.106173	0.15	1	0.49
CL3	0.935077728	C_20 H_20 O_2 S_1	323.111223	0.1	1	0.5
CL3	0.892376179	C_23 H_24 O_3 S_1	379.137431	0.13	1.043	0.46
CL3	0.814885073	C_19 H_18 O_3 S_1	325.090494	0.158	0.947	0.52
CL3	0.779550298	C_19 H_18 O_2 S_1	309.095557	0.105	0.947	0.53
CL3	0.765316833	C_18 H_16 O_3 S_1	311.074845	0.167	0.889	0.55
CL3	0.739550239	C_18 H_16 O_2 S_1	295.079926	0.111	0.889	0.56
CL3	0.716485444	C_22 H_22 O_4 S_1	381.116749	0.182	1	0.47
CL3	0.663491822	C_21 H_20 O_4 S_1	367.101059	0.19	0.952	0.5
CL3	0.592639726	C_20 H_32 O_3	319.22789	0.15	1.6	0.19
CL3	0.5713909	C_21 H_20 O_3 S_1	351.106156	0.143	0.952	0.51
CL3	0.532404174	C_20 H_18 O_3 S_1	337.090495	0.15	0.9	0.54
CL3	0.48846856	C_10 H_18 O_2	169.1234	0.2	1.8	0.11
CL3	0.482266598	C_10 H_18 O_1	153.128497	0.1	1.8	0.16
CL3	0.471147405	C_19 H_34 O_6	357.228264	0.316	1.789	0
CL3	0.447964397	C_17 H_30 O_5	313.202053	0.294	1.765	0.03
CL3	0.447750778	C_12 H_22 O_4	229.144532	0.333	1.833	0
CL3	0.433373944	C_19 H_32 O_7	371.207547	0.368	1.684	0.03
CL3	0.427650904	C_9 H_16 O_1	139.112842	0.111	1.778	0.18
CL3	0.424739223	C_19 H_32 O_6	355.212603	0.316	1.684	0.06
CL3	0.422334114	C_18 H_30 O_6	341.196968	0.333	1.667	0.07
CL3	0.42011097	C_14 H_24 O_5	271.155119	0.357	1.714	0.04
CL3	0.418341627	C_11 H_20 O_2	183.139044	0.182	1.818	0.1
CL3	0.417010569	C_18 H_30 O_7	357.191851	0.389	1.667	0.03
CL3	0.414451732	C_18 H_32 O_6	343.212632	0.333	1.778	0
CL3	0.413661418	C_17 H_30 O_6	329.196942	0.353	1.765	0
CL3	0.40877866	C_16 H_28 O_5	299.186396	0.312	1.75	0.04
CL3	0.405147374	C_17 H_28 O_6	327.181331	0.353	1.647	0.07
CL3	0.391624071	C_20 H_34 O_7	385.223196	0.35	1.7	0.03
CL3	0.383951441	C_15 H_26 O_4	269.175832	0.267	1.733	0.08
CL3	0.383836445	C_19 H_32 O_5	339.217677	0.263	1.684	0.09
CL3	0.381670728	C_15 H_24 O_6	299.150034	0.4	1.6	0.08
CL3	0.380574151	C_16 H_28 O_4	283.191472	0.25	1.75	0.07
CL3	0.379140455	C_20 H_34 O_6	369.228289	0.3	1.7	0.06
CL3	0.377976935	C_17 H_28 O_7	343.176241	0.412	1.647	0.04
CL3	0.373619698	C_16 H_26 O_6	313.165666	0.375	1.625	0.08
CL3	0.37227288	C_11 H_18 O_2	181.123407	0.182	1.636	0.2

CL3	0.369799743	C_13 H_22 O_3	225.149612	0.231	1.692	0.13
CL3	0.365788715	C_14 H_22 O_6	285.134359	0.429	1.571	0.09
CL3	0.365260778	C_19 H_32 O_8	387.202431	0.421	1.684	0
CL3	0.359804858	C_17 H_28 O_5	311.186421	0.294	1.647	0.1
CL3	0.359631426	C_18 H_30 O_5	325.202037	0.278	1.667	0.1
CL3	0.358878011	C_16 H_26 O_4	281.175841	0.25	1.625	0.14
CL3	0.353407629	C_19 H_30 O_6	353.196972	0.316	1.579	0.12
CL3	0.352642715	C_13 H_20 O_6	271.118698	0.462	1.538	0.1
CL3	0.351128789	C_14 H_22 O_5	269.139459	0.357	1.571	0.13
CL3	0.346236169	C_17 H_28 O_8	359.171167	0.471	1.647	0
CL3	0.343746193	C_10 H_16 O_2	167.107744	0.2	1.6	0.22
CL3	0.342571318	C_16 H_26 O_5	297.17074	0.312	1.625	0.11
CL3	0.34237682	C_17 H_28 O_4	295.191502	0.235	1.647	0.13
CL3	0.340715726	C_14 H_22 O_4	253.144544	0.286	1.571	0.17
CL3	0.340687547	C_12 H_18 O_4	225.113222	0.333	1.5	0.2
CL3	0.34052212	C_19 H_30 O_7	369.191875	0.368	1.579	0.1
CL3	0.337429612	C_18 H_28 O_6	339.18131	0.333	1.556	0.13
CL3	0.337340196	C_12 H_20 O_3	211.133954	0.25	1.667	0.14
CL3	0.337334762	C_15 H_24 O_5	283.155118	0.333	1.6	0.12
CL3	0.334228353	C_13 H_20 O_4	239.128891	0.308	1.538	0.18
CL3	0.333047875	C_15 H_24 O_4	267.160202	0.267	1.6	0.15
CL3	0.332838601	C_14 H_24 O_3	239.165282	0.214	1.714	0.12
CL3	0.329684322	C_13 H_20 O_5	255.123779	0.385	1.538	0.14
CL3	0.328772923	C_16 H_24 O_6	311.150029	0.375	1.5	0.15
CL3	0.327769008	C_17 H_26 O_6	325.165665	0.353	1.529	0.14
CL3	0.325684444	C_11 H_16 O_4	211.097582	0.364	1.455	0.22
CL3	0.323893871	C_19 H_30 O_8	385.186768	0.421	1.579	0.07
CL3	0.322368097	C_20 H_32 O_8	399.202474	0.4	1.6	0.06
CL3	0.31993028	C_12 H_18 O_5	241.108149	0.417	1.5	0.16
CL3	0.317804735	C_18 H_28 O_7	355.176209	0.389	1.556	0.1
CL3	0.316690584	C_18 H_28 O_8	371.171119	0.444	1.556	0.07
CL3	0.314488359	C_15 H_24 O_7	315.144911	0.467	1.6	0.04
CL3	0.311764146	C_20 H_32 O_7	383.207547	0.35	1.6	0.09
CL3	0.3100008	C_17 H_26 O_8	357.155505	0.471	1.529	0.08
CL3	0.307979068	C_14 H_22 O_7	301.129279	0.5	1.571	0.05
CL3	0.307657978	C_23 H_38 O_8	441.249361	0.348	1.652	0.05
CL3	0.304721238	C_17 H_26 O_5	309.170731	0.294	1.529	0.17
CL3	0.300984134	C_21 H_32 O_8	411.202452	0.381	1.524	0.12
CL3	0.299489239	C_23 H_36 O_8	439.233718	0.348	1.565	0.11

CL3	0.299008798	C_17 H_26 O_7	341.160558	0.412	1.529	0.11
CL3	0.297588729	C_16 H_24 O_7	327.144929	0.438	1.5	0.12
CL3	0.297098437	C_22 H_34 O_8	425.218113	0.364	1.545	0.11
CL3	0.291115675	C_18 H_28 O_5	323.186378	0.278	1.556	0.16
CL3	0.290891244	C_16 H_24 O_5	295.155101	0.312	1.5	0.19
CL3	0.289711477	C_22 H_34 O_7	409.223187	0.318	1.545	0.14
CL3	0.285751946	C_20 H_30 O_8	397.186805	0.4	1.5	0.12
CL3	0.28380035	C_14 H_20 O_6	283.118694	0.429	1.429	0.18
CL3	0.282500123	C_22 H_34 O_9	441.213029	0.409	1.545	0.09
CL3	0.28199754	C_15 H_22 O_7	313.129287	0.467	1.467	0.13
CL3	0.281072573	C_19 H_28 O_8	383.171122	0.421	1.474	0.13
CL3	0.277503801	C_19 H_30 O_9	401.181709	0.474	1.579	0.03
CL3	0.277407445	C_22 H_32 O_8	423.202415	0.364	1.455	0.17
CL3	0.275801673	C_18 H_26 O_8	369.155479	0.444	1.444	0.14
CL3	0.265359879	C_22 H_32 O_9	439.197378	0.409	1.455	0.14
CL3	0.261235615	C_21 H_30 O_8	409.186765	0.381	1.429	0.18
CL4	1	C_6 H_14 O_3 S_1	165.059088	0.5	2.333	0
CL4	1	C_6 H_4 O_4 S_1	170.975745	0.667	0.667	0.67
CL4	1	C_7 H_14 O_3 S_1	177.059077	0.429	2	0
CL4	1	C_7 H_16 O_3 S_1	179.074743	0.429	2.286	0
CL4	1	C_9 H_18 O_3 S_1	205.090435	0.333	2	0
CL4	1	C_11 H_20 O_3 S_1	231.106043	0.273	1.818	0
CL4	1	C_10 H_19 O_5 N_1 S_1	264.091193	0.5	1.9	0
CL4	1	C_9 H_18 O_6 S_2	285.047161	0.667	2	0
CL4	1	C_12 H_24 O_8 S_1	327.111894	0.667	2	0
CL4	1	C_14 H_25 O_6 N_1 S_1	334.132943	0.429	1.786	0
CL4	1	C_14 H_27 O_6 N_1 S_1	336.148648	0.429	1.929	0
CL4	1	C_12 H_21 O_8 N_1 S_1	338.091577	0.667	1.75	0
CL4	1	C_13 H_25 O_7 N_1 S_1	338.127827	0.538	1.923	0
CL4	1	C_12 H_22 O_9 S_1	341.091213	0.75	1.833	0
CL4	1	C_14 H_25 O_7 N_1 S_1	350.127906	0.5	1.786	0
CL4	1	C_14 H_27 O_7 N_1 S_1	352.143548	0.5	1.929	0
CL4	1	C_15 H_27 O_7 N_1 S_1	364.143515	0.467	1.8	0
CL4	1	C_16 H_29 O_7 N_1 S_1	378.159186	0.438	1.812	0
CL4	1	C_16 H_29 O_8 N_1 S_1	394.154092	0.5	1.812	0
CL4	1	C_17 H_29 O_8 N_1 S_1	406.154161	0.471	1.706	0
CL4	1	C_16 H_30 O_8 S_2	413.130784	0.5	1.875	0
CL4	1	C_16 H_30 O_10 S_1	413.148742	0.625	1.875	0
CL4	1	C_16 H_27 O_10 N_1 S_1	424.128283	0.625	1.688	0

CL4	1	C_17 H_32 O_10 S_1	427.164287	0.588	1.882	0
CL4	1	C_17 H_32 O_9 S_2	443.141312	0.529	1.882	0
CL4	1	C_20 H_33 O_8 N_1 S_1	446.185299	0.4	1.65	0
CL4	1	C_17 H_27 O_11 N_1 S_1	452.123186	0.647	1.588	0
CL4	1	C_21 H_33 O_9 N_1 S_1	474.180325	0.429	1.571	0
CL4	1	C_22 H_29 O_9 N_1 S_1	482.148809	0.409	1.318	0.16
CL4	1	C_19 H_29 O_12 N_1 S_1	494.133745	0.632	1.526	0
CL4	1	C_20 H_33 O_11 N_1 S_1	494.170139	0.55	1.65	0
CL4	1	C_20 H_36 O_12 S_1	499.185334	0.6	1.8	0
CL4	1	C_21 H_35 O_11 N_1 S_1	508.185964	0.524	1.667	0
CL4	1	C_20 H_33 O_12 N_1 S_1	510.164993	0.6	1.65	0
CL4	1	C_22 H_40 O_11 S_1	511.22162	0.5	1.818	0
CL4	1	C_21 H_38 O_12 S_1	513.201011	0.571	1.81	0
CL4	1	C_22 H_35 O_11 N_1 S_1	520.18586	0.5	1.591	0
CL4	1	C_22 H_38 O_12 S_1	525.201062	0.545	1.727	0
CL4	1	C_24 H_42 O_11 S_1	537.237369	0.458	1.75	0
CL4	1	C_23 H_40 O_12 S_1	539.216686	0.522	1.739	0
CL4	1	C_24 H_37 O_11 N_1 S_1	546.201586	0.458	1.542	0
CL4	1	C_23 H_40 O_13 S_1	555.21159	0.565	1.739	0
CL4	1	C_24 H_37 O_12 N_1 S_1	562.196393	0.5	1.542	0
CL4	1	C_16 H_38 O_16 N_6	569.227031	1	2.375	0
CL4	1	C_25 H_42 O_13 S_1	581.22743	0.52	1.68	0
CL4	1	C_26 H_44 O_13 S_1	595.243089	0.5	1.692	0
CL4	1	C_25 H_42 O_14 S_1	597.222128	0.56	1.68	0
CL4	1	C_28 H_48 O_12 S_1	607.279383	0.429	1.714	0
CL4	1	C_27 H_44 O_14 S_1	623.23784	0.519	1.63	0
CL4	1	C_29 H_46 O_14 S_1	649.253426	0.483	1.586	0
CL4	0.983766331	C_5 H_4 O_2 S_1	126.985899	0.4	0.8	0.67
CL4	0.983725335	C_13 H_24 O_7 S_2	355.089089	0.538	1.846	0
CL4	0.982912669	C_10 H_20 O_5	219.123801	0.5	2	0
CL4	0.981794467	C_21 H_38 O_11 S_1	497.206009	0.524	1.81	0
CL4	0.981645892	C_10 H_18 O_3 S_1	217.090383	0.3	1.8	0
CL4	0.979901385	C_11 H_22 O_4 S_1	249.116651	0.364	2	0
CL4	0.979620384	C_13 H_24 O_4 S_1	275.132224	0.308	1.846	0
CL4	0.977910078	C_13 H_24 O_6 S_2	339.094169	0.462	1.846	0
CL4	0.977312015	C_16 H_32 O_8 S_1	383.174467	0.5	2	0
CL4	0.976867926	C_17 H_30 O_9 S_2	441.125737	0.529	1.765	0
CL4	0.976743522	C_14 H_26 O_6 S_2	353.109743	0.429	1.857	0
CL4	0.975895668	C_19 H_31 O_8 N_1 S_1	432.169766	0.421	1.632	0

CL4	0.975813663	C_13 H_23 O_6 N_1 S_1	320.117307	0.462	1.769	0
CL4	0.975233975	C_21 H_33 O_11 N_1 S_1	506.170225	0.524	1.571	0
CL4	0.974712397	C_6 H_12 O_4 S_1	179.038358	0.667	2	0
CL4	0.973940439	C_17 H_29 O_7 N_1 S_1	390.159191	0.412	1.706	0
CL4	0.973361797	C_14 H_26 O_8 S_2	385.099601	0.571	1.857	0
CL4	0.973320085	C_18 H_34 O_10 S_1	441.179942	0.556	1.889	0
CL4	0.972745009	C_24 H_42 O_12 S_1	553.232293	0.5	1.75	0
CL4	0.972698606	C_20 H_38 O_9 S_1	453.216372	0.45	1.9	0
CL4	0.972456508	C_17 H_30 O_8 S_2	425.130826	0.471	1.765	0
CL4	0.972447498	C_22 H_35 O_10 N_1 S_1	504.190653	0.455	1.591	0
CL4	0.972270542	C_14 H_26 O_7 S_2	369.104681	0.5	1.857	0
CL4	0.971504606	C_27 H_46 O_21	705.245673	0.778	1.704	0
CL4	0.970285299	C_20 H_40 O_18 N_6	651.232472	0.9	2	0
CL4	0.970272731	C_21 H_33 O_12 N_1 S_1	522.165144	0.571	1.571	0
CL4	0.970037373	C_20 H_34 O_7 N_2	413.229286	0.35	1.7	0
CL4	0.969496977	C_12 H_22 O_4 S_1	261.116589	0.333	1.833	0
CL4	0.969066849	C_27 H_46 O_10 S_1	561.273831	0.37	1.704	0
CL4	0.968863246	C_13 H_24 O_12 S_1	403.091625	0.923	1.846	0
CL4	0.968405432	C_22 H_35 O_12 N_1 S_1	536.180894	0.545	1.591	0
CL4	0.967428955	C_9 H_9 O_6 N_1 S_1	258.007747	0.667	1	0.25
CL4	0.966888962	C_15 H_28 O_10 S_1	399.133038	0.667	1.867	0
CL4	0.966548524	C_16 H_28 O_8 S_2	411.115228	0.5	1.75	0
CL4	0.965969053	C_13 H_24 O_9 S_2	387.079024	0.692	1.846	0
CL4	0.965491023	C_20 H_33 O_9 N_1 S_1	462.180284	0.45	1.65	0
CL4	0.965037615	C_16 H_28 O_10 S_2	443.105044	0.625	1.75	0
CL4	0.962649387	C_9 H_20 O_5 S_1	239.095879	0.556	2.222	0
CL4	0.961911411	C_26 H_44 O_12 S_1	579.247913	0.462	1.692	0
CL4	0.961776322	C_23 H_35 O_10 N_1 S_1	516.190767	0.435	1.522	0
CL4	0.958264569	C_13 H_22 O_11 S_1	385.081042	0.846	1.692	0
CL4	0.957277292	C_12 H_22 O_6 S_2	325.078467	0.5	1.833	0
CL4	0.956701577	C_16 H_28 O_7 S_2	395.120238	0.438	1.75	0
CL4	0.953209579	C_14 H_23 O_9 N_1 S_1	380.102006	0.643	1.643	0
CL4	0.952769405	C_23 H_38 O_14 S_1	569.190876	0.609	1.652	0
CL4	0.952307369	C_8 H_14 O_3 S_1	189.05906	0.375	1.75	0
CL4	0.952253821	C_15 H_26 O_10 S_2	429.089488	0.667	1.733	0
CL4	0.951897097	C_15 H_25 O_7 N_1 S_1	362.127874	0.467	1.667	0
CL4	0.951198996	C_20 H_36 O_11 S_1	483.190499	0.55	1.8	0
CL4	0.951176632	C_15 H_25 O_9 N_1 S_1	394.117772	0.6	1.667	0
CL4	0.951126238	C_23 H_40 O_9 S_1	491.231937	0.391	1.739	0

CL4	0.950911649	C_11 H_20 O_6 S_2	311.062834	0.545	1.818	0
CL4	0.950894521	C_21 H_36 O_13 S_1	527.18034	0.619	1.714	0
CL4	0.950538278	C_18 H_34 O_9 S_1	425.185017	0.5	1.889	0
CL4	0.94980146	C_22 H_40 O_10 S_1	495.226941	0.455	1.818	0
CL4	0.949656586	C_19 H_33 O_9 N_1 S_1	450.180343	0.474	1.737	0
CL4	0.948807597	C_6 H_12 O_7 S_1	227.023096	1.167	2	0
CL4	0.947685794	C_24 H_42 O_10 S_1	521.24245	0.417	1.75	0
CL4	0.946650046	C_19 H_36 O_9 S_1	439.200733	0.474	1.895	0
CL4	0.946198912	C_9 H_16 O_3 S_1	203.074765	0.333	1.778	0
CL4	0.945983828	C_21 H_38 O_10 S_1	481.211205	0.476	1.81	0
CL4	0.945578809	C_14 H_26 O_5 S_1	305.142773	0.357	1.857	0
CL4	0.944419323	C_14 H_28 O_8 S_1	355.143277	0.571	2	0
CL4	0.944230627	C_11 H_22 O_6 S_2	313.07844	0.545	2	0
CL4	0.943829671	C_20 H_32 O_7 N_2	411.213676	0.35	1.6	0.03
CL4	0.943014723	C_17 H_32 O_8	363.202445	0.471	1.882	0
CL4	0.94148207	C_19 H_31 O_11 N_1 S_1	480.154474	0.579	1.632	0
CL4	0.940727514	C_15 H_30 O_8 S_1	369.158824	0.533	2	0
CL4	0.939416926	C_17 H_27 O_10 N_1 S_1	436.128298	0.588	1.588	0
CL4	0.938214225	C_8 H_16 O_8 S_1	271.049302	1	2	0
CL4	0.938124358	C_12 H_21 O_5 N_1 S_1	290.106745	0.417	1.75	0
CL4	0.934426175	C_13 H_21 O_9 N_1 S_1	366.086433	0.692	1.615	0
CL4	0.934232997	C_18 H_36 O_18 N_6	623.201138	1	2	0
CL4	0.933886979	C_18 H_29 O_8 N_1 S_1	418.154246	0.444	1.611	0
CL4	0.933632397	C_13 H_21 O_5 N_1 S_1	302.106738	0.385	1.615	0
CL4	0.933269051	C_13 H_22 O_8 S_2	369.068357	0.615	1.692	0
CL4	0.931914605	C_15 H_23 O_5 N_1 S_1	328.122434	0.333	1.533	0.05
CL4	0.931842831	C_15 H_23 O_10 N_1 S_1	408.096962	0.667	1.533	0
CL4	0.930634348	C_27 H_44 O_13 S_1	607.242837	0.481	1.63	0
CL4	0.930010241	C_22 H_38 O_13 S_1	541.195956	0.591	1.727	0
CL4	0.928087462	C_22 H_33 O_10 N_1 S_1	502.175154	0.455	1.5	0
CL4	0.923107505	C_14 H_25 O_8 N_1 S_1	366.122884	0.571	1.786	0
CL4	0.921308591	C_23 H_40 O_11 S_1	523.221793	0.478	1.739	0
CL4	0.921054254	C_7 H_12 O_8 S_1	255.018027	1.143	1.714	0
CL4	0.918865649	C_11 H_19 O_7 N_1 S_1	308.080942	0.636	1.727	0
CL4	0.913678018	C_16 H_26 O_9 S_2	425.094473	0.562	1.625	0
CL4	0.912450844	C_26 H_42 O_14 S_1	609.222146	0.538	1.615	0
CL4	0.912323557	C_22 H_31 O_10 N_1 S_1	500.159494	0.455	1.409	0.07
CL4	0.9101721	C_11 H_22 O_5	233.13939	0.455	2	0
CL4	0.907197467	C_17 H_30 O_12 S_1	457.138372	0.706	1.765	0

CL4	0.906543071	C_16 H_18 O_9 N_4	409.099899	0.562	1.125	0.2
CL4	0.903359039	C_11 H_16 O_13 N_6	439.070358	1.182	1.455	0
CL4	0.899870309	C_22 H_36 O_7 S_1	443.210779	0.318	1.636	0.03
CL4	0.898682247	C_19 H_34 O_11 S_1	469.1748	0.579	1.789	0
CL4	0.897895406	C_20 H_31 O_9 N_1 S_1	460.164659	0.45	1.55	0
CL4	0.896913077	C_14 H_23 O_8 N_1 S_1	364.107157	0.571	1.643	0
CL4	0.895492947	C_7 H_14 O_4 S_1	193.053988	0.571	2	0
CL4	0.892695124	C_17 H_30 O_11 S_1	441.143538	0.647	1.765	0
CL4	0.892314406	C_12 H_19 O_8 N_1 S_1	336.075859	0.667	1.583	0
CL4	0.89079874	C_21 H_38 O_8 S_1	449.221426	0.381	1.81	0
CL4	0.885785067	C_18 H_36 O_5 N_1 P_1	376.225974	0.278	2	0
CL4	0.88453583	C_27 H_44 O_12 S_1	591.247963	0.444	1.63	0
CL4	0.883790397	C_20 H_36 O_7 S_1	419.210878	0.35	1.8	0
CL4	0.883180491	C_23 H_33 O_11 N_1 S_1	530.170072	0.478	1.435	0.03
CL4	0.883118963	C_20 H_31 O_11 N_1 S_1	492.154513	0.55	1.55	0
CL4	0.881747771	C_11 H_18 O_5 S_2	293.052253	0.455	1.636	0
CL4	0.878347908	C_21 H_34 O_6 S_1	413.200534	0.286	1.619	0.06
CL4	0.877478058	C_20 H_31 O_12 N_1 S_1	508.149452	0.6	1.55	0
CL4	0.877051798	C_18 H_34 O_8 S_1	409.19012	0.444	1.889	0
CL4	0.87554862	C_22 H_33 O_11 N_1 S_1	518.170198	0.5	1.5	0
CL4	0.872837948	C_18 H_32 O_12 S_1	471.154037	0.667	1.778	0
CL4	0.871643461	C_14 H_21 O_6 N_1 S_1	330.101719	0.429	1.5	0
CL4	0.865331177	C_13 H_26 O_6 S_1	309.137724	0.462	2	0
CL4	0.859966937	C_23 H_28 O_13 N_4	567.158103	0.565	1.217	0.12
CL4	0.853532182	C_19 H_34 O_12 S_1	485.169736	0.632	1.789	0
CL4	0.851918978	C_16 H_32 O_7 S_1	367.179625	0.438	2	0
CL4	0.850186149	C_23 H_38 O_12 S_1	537.201122	0.522	1.652	0
CL4	0.84811872	C_25 H_40 O_14 S_1	595.206552	0.56	1.6	0
CL4	0.844219857	C_10 H_18 O_8 S_1	297.064979	0.8	1.8	0
CL4	0.843164055	C_14 H_21 O_9 N_1 S_1	378.086459	0.643	1.5	0
CL4	0.842243642	C_21 H_31 O_12 N_1 S_1	520.149479	0.571	1.476	0
CL4	0.840988621	C_18 H_32 O_6 S_1	375.184722	0.333	1.778	0
CL4	0.838922703	C_18 H_34 O_7 S_1	393.195261	0.389	1.889	0
CL4	0.83780887	C_24 H_38 O_14 S_1	581.190757	0.583	1.583	0
CL4	0.836334842	C_26 H_40 O_14 S_1	607.206431	0.538	1.538	0
CL4	0.832674995	C_26 H_37 O_11 N_3	566.235621	0.423	1.423	0.09
CL4	0.832147109	C_23 H_36 O_14 S_1	567.17531	0.609	1.565	0
CL4	0.830857071	C_24 H_40 O_9 S_1	503.23189	0.375	1.667	0
CL4	0.827848153	C_27 H_42 O_14 S_1	621.222099	0.519	1.556	0

CL4	0.818246006	C_13 H_22 O_4 S_1	273.116587	0.308	1.692	0
CL4	0.815280299	C_13 H_22 O_10 S_1	369.086077	0.769	1.692	0
CL4	0.814617107	C_15 H_27 O_7 P_1	349.142314	0.467	1.8	0
CL4	0.80559371	C_21 H_31 O_10 N_1 S_1	488.159499	0.476	1.476	0
CL4	0.802404391	C_10 H_16 O_3 S_1	215.074729	0.3	1.6	0.07
CL4	0.797874447	C_20 H_29 O_11 N_1 S_1	490.138878	0.55	1.45	0
CL4	0.7928563	C_13 H_26 O_7 S_1	325.132673	0.538	2	0
CL4	0.789534614	C_20 H_34 O_13 S_1	513.164729	0.65	1.7	0
CL4	0.764731408	C_19 H_32 O_6 S_1	387.184744	0.316	1.684	0
CL4	0.762268803	C_17 H_23 O_9 N_1 S_1	416.102097	0.529	1.353	0.05
CL4	0.752479005	C_12 H_20 O_4 S_1	259.100974	0.333	1.667	0
CL4	0.729026473	C_17 H_25 O_10 N_1 S_1	434.112668	0.588	1.471	0
CL4	0.728267186	C_15 H_23 O_7 N_1 S_1	360.11226	0.467	1.533	0
CL4	0.718331926	C_17 H_23 O_8 N_1 S_1	400.107288	0.471	1.353	0.09
CL4	0.708915722	C_20 H_32 O_13 S_1	511.149138	0.65	1.6	0
CL4	0.695070649	C_17 H_28 O_12 S_1	455.122804	0.706	1.647	0
CL4	0.655440067	C_27 H_40 O_13 S_1	603.211643	0.481	1.481	0.03
CL4	0.629307792	C_27 H_35 O_12 N_3	592.214845	0.444	1.296	0.17
CL4	0.601626981	C_11 H_16 O_5 S_1	259.064549	0.455	1.455	0.07
CL4	0.408295443	C_11 H_20 O_6	247.118725	0.545	1.818	0
CL4	0.385459831	C_8 H_12 O_6	203.056114	0.75	1.5	0
CL4	0.376415548	C_10 H_8 O_4	191.034989	0.4	0.8	0.62
CL4	0.30989565	C_9 H_13 O_4 N_1	198.077172	0.444	1.444	0.17
CL4	0.296192425	C_8 H_9 O_4 N_1	182.045894	0.5	1.125	0.4
CL4	0.26789301	C_18 H_23 O_8 N_3	408.141227	0.444	1.278	0.18
CL4	0.265005359	C_18 H_23 O_7 N_3	392.146324	0.389	1.278	0.22
